# Overexpression of *SlRBZ* Results in Chlorosis and Dwarfism through Impairing Chlorophyll, Carotenoid, and Gibberellin Biosynthesis in Tomato

**DOI:** 10.3389/fpls.2016.00907

**Published:** 2016-06-22

**Authors:** Mingqin Fan, Shenghua Gao, Junling Ren, Qihong Yang, Hanxia Li, Changxian Yang, Zhibiao Ye

**Affiliations:** ^1^Key Laboratory of Horticultural Plant Biology (MOE), Key Laboratory of Horticultural Crop Biology and Genetic Improvement (Central Region), MOA, Huazhong Agricultural UniversityWuhan, China; ^2^School of Biology and Food Engineering, Fuyang Teachers CollegeFuyang, China

**Keywords:** *SlRBZ*, chlorosis, dwarfism, chloroplast, tomato

## Abstract

ZFPs play important roles in many biological processes, including plant development, stress response, and phytohormone response. RanBP2-type zinc finger transcription factors have been characterized in animals and humans. However, their functions remain largely unknown in plants. In this study, we identified a RanBP2-type zinc finger protein gene (*SlRBZ*) in tomato. *SlRBZ* was constitutively expressed in roots, stems, leaves, flowers, and fruits. The SlRBZ-GFP fused protein was localized in the nucleus. Overexpression of *SlRBZ* resulted in chlorosis and dwarf phenotypes in tomato. Determination of physiological index showed that chlorophyll, carotenoid, and GAs contents were evidently decreased in transgenic plants. Furthermore, the qRT-PCR and RNA-Seq analyses demonstrated that the transcription of the genes involved in these biosynthesis pathways obviously decreased in *SlRBZ*-OE plants. In addition, ultrastructural observation by transmission electron microscopy indicated that plastids could not develop into mature chloroplasts with normal chloroplast membrane and thylakoid membrane system in *SlRBZ*-OE plants. The results suggest that overexpression of *SlRBZ* may impair the biosynthesis of chlorophyll, carotenoid, and gibberellin through blocking chloroplast development, resulting in chlorosis and dwarfism in tomato.

## Introduction

Chlorophyll (Chl) has important function in light harvesting and photosynthetic energy transduction, which is closely related to chloroplast-nuclear signaling and chloroplast development (Eckhardt et al., 2004). Chl biosynthesis is a complex process, including the formation of 5-aminolevulinic acid (ALA), protoporphyrin IX from eight molecules of ALA, and Chl in the magnesium branch (Beale, 1999; Eckhardt et al., 2004). A limiting factor of the whole pathway is glutamyl tRNA reductase (GluTR), which is the key enzyme for ALA formation. *HEMA1, HEMA2*, and *HEMA 3* encode GluTR and are expressed in different tissues and environmental conditions (McCormac et al., 2001). Eight molecules of ALA are first converted into uroporphyrinogen III. After three steps, uroporphyrinogen III is oxidized into Proto under the function of protoporphyrinogen oxidase (Tanaka and Tanaka, 2007). Mg–chelatase consists of CHLI, CHLD, and CHLH (Papenbrock et al., 1997). Under the combined effect of these three enzymes, MgProtoMe cyclase, NADPH-Pchlide oxidoreductase, and Chl synthase, Proto is transferred into Chl (Eckhardt et al., 2004). Inhibition of each enzymatic step can affect the synthesis of Chl and result in leaf color change. The transcription of these enzymes required for Chl biosynthesis is individually and independently regulated. Meanwhile, light and developmental processes could induce the expression of the majority of these enzymes. Chloroplast formation influences leaf color in plants. Many chloroplastid proteins can partially block chloroplast development and result in a chlorotic phenotype (Carol et al., 1999; Wu et al., 2007). Chloroplasts are arrested and Chl is almost absent in the insertion mutant of the *HST* gene, which encodes an important enzyme participating in the biosynthesis of the PSII mobile electron transport co-factor PQ (Chao et al., 2014).

Gibberellins (GAs) are important for plant growth and development. Decreased GA content can cause plant dwarfism. Many different isoforms of GAs exist in nature, whereas only a few of them possess biological activity and regulate plant development, including GA1, GA3, GA4, and GA7 (Hedden and Phillips, 2000). GA12 and GA53 as precursors can be converted into many GA intermediates and bioactive GAs, wherein GA 20-oxidases (GA20ox) and GA 3-oxidases (GA3ox) have key roles (Ross et al., 1999). In addition, GA3ox catalyzes the final step in the synthesis of bioactive GAs. However, GA 2-oxidase (GA2ox) functions in converting active GAs and their precursors into inactive isoforms (Thomas et al., 1999). The mechanisms of GA biosynthesis and catabolism have been well-characterized in plants. As reported, GAs are synthesized from the common diterpene precursor geranylgeranyl diphosphate (GGPP) in plastids (Okada et al., 2000). During this process, GGPP is first separately converted into ent-kaurene by ent-copalyl diphosphate synthase (CPS) and ent-kaurene synthase (KS; Duncan and West, 1981). Then, the ent-kaurene is converted into GA12 by ent-kaurene oxidase (KO) and ent-kaurenoic oxidase (KAO; Yamaguchi, 2008). Furthermore, GGPP is also the precursor necessary for carotenoids and Chl biosynthesis (Rodríguez-Concepción et al., 2001). Overexpression of *PSY* results in dwarfism and chlorosis in tomato, as it is the first gene for a carotenoid biosynthetic enzyme. Chl, GA, and carotenoid are synthesized in plastids, most of which are controlled by nuclear genes (Fray et al., 1995).

Chloroplasts are organelles that differentiate from plastids in plant cells (Gruissem, 1989). Different species, tissues, and environmental conditions can influence their ultimate shape and structure (Izawa and Good, 1966; Dodge, 1970). Notably, chloroplasts are surrounded by outer and inner envelope membranes. Chloroplast biogenesis is coordinately controlled by the proteins encoded in the nuclear and plastid genomes (Chen and Schnell, 1999). Moreover, ~80% of chloroplast proteins are encoded by nuclear genes (Ohyama et al., 1986). These proteins participate in protein transport, translation, and folding during chloroplast development (Klein and Mullet, 1986; Hendrick and Hartl, 1993; Cline and Henry, 1996; Fuks and Schnell, 1997). Many genes involved in the chloroplast development have been isolated by analyzing *Arabidopsis* mutants. A nuclear-encoded sigma factor, *AtSIG6* controls early chloroplast development in *Arabidopsis* cotyledons, the mutation of which results in a cotyledon-specific pale green phenotype (Ishizaki et al., 2005). Co-suppression of two highly conserved heat shock protein genes, namely, *cpHsc70-1* and *cpHsc70-2*, causes a white and stunted phenotype (Latijnhouwers et al., 2010). Huang et al. (2009) uncovered that the *EMB1303* gene is required for chloroplast development, which encodes a chloroplast-localized protein in *Arabidopsis* (Huang et al., 2009). The *Arabidopsis MDA1* gene affects chloroplast morphology and *mda1* mutants exhibit reduced pigmentation of cotyledons, leaves, stems, and sepals (Robles et al., 2012). Moreover, a guanylate kinase encoded by a nuclear gene *VIRESCENT 2* participates in chloroplast differentiation in rice (Sugimoto et al., 2007). Impairment of chloroplast development brings about chlorophyll (Chl) reduction.

Zinc finger protein (ZFP) was initially discovered in *Xenopus* oocytes (Miller et al., 1985). And many ZFPs have been identified in eukaryotes. Previous studies demonstrated that ZFPs participate in many biological processes, including plant growth and development, stress response, and phytohormone response (Laity et al., 2001). Ran-binding proteins (RanBPs), a novel type of zinc finger transcription factors, also broadly existed in higher eukaryotes. RanBP ZFP has three Ran-binding domains and two zinc finger motifs, which is a component of the nuclear pore complex and important in nuclear pore function (Chang et al., 2007). Further studies have demonstrated that RanBP2 complex is required for E3 activity (Werner et al., 2012). In humans, RanBP ZFPs have been implicated in the regulation of mRNA processing. However, little is known about the functions of RanBP ZFPs in plants. To date, only one gene of this type has been characterized in cotton, *RanBP2 ZFP*, which has been identified to participate in the different development stages of glands and may function in the development of the cotton gland (Chang et al., 2007). Therefore, the characterization of the functions of RanBP ZFPs in plants will shed new insights into the roles of these transcription factors in developmental processes.

In this study, the *SlRBZ* gene was characterized as RanBP-type transcription factor, which was localized in the nucleus. Overexpression of *SlRBZ* resulted in chlorosis and dwarfism phenotypes in tomato. The synthesis of Chl, carotenoid and GA, as well as the expression levels of genes related to these pathways, were evidently suppressed in *SlRBZ*-OE plants. Meanwhile, ultrastructural observation revealed that chloroplast formation was blocked. This conclusion was further supported by RNA-Seq analyses and qRT-PCR. These results demonstrate that overexpression of *SlRBZ* may inhibit the biosynthesis of Chl, carotenoid, and GA through affecting chloroplast formation, resulting in chlorosis and dwarfism.

## Materials and methods

### Plant materials and treatments

Tomato (*Solanum lycopersicum* cv. Ailsa Craig) plants and transgenic lines in this background were grown in a growth chamber (16 h light/8 h dark photoperiod; 25°C). In order to investigate the response of *SlRBZ* gene to different growth regulator, the four-leaf-stage seedlings were separately sprayed with 100 μM GA_3_, 100 μM IAA, and 100 μM ABA. Tissues were collected from the roots, stems, leaves, flowers, and fruits of AC plants for analyzing the expression pattern of *SlRBZ*. Dwarf phenotypes of transgenic plants were restored by spraying with 100 μM GA_3_ at an interval of 3 d. Experiments were carried out with three independent biological replicates per treatment (each group with eight plants). The seedlings treated with distilled water were used as controls.

### Isolation of *SlRBZ* gene, construction of expression vectors, and transgenic analysis

The full-length coding sequence of tomato *SlRBZ* gene was amplified from the AC cDNA using gene-specific primers (forward primer: 5′-GCTCTAGA ATGGGACGAGAAGGAGATTG-3′; reverse primer: 5′-GCGGTACC CTATGCAACAGTAACAGGTTGAGT-3′) based on the gene sequence (Solyc03g033560). The fragment of the *SlRBZ* gene was inserted into the pMV2 vector under control of the CaMV 35S promoter. The RNAi vector was constructed by amplifying a 116 bp fragment and ligating with pHellsgate 2 through BP reaction. Both constructs were verified by double digestion and transformed into AC mediated by *Agrobacterium tumefaciens* strain C58. The transgenic plants were further detected with PCR using genomic DNA as templates and CaMV35S forward and gene-specific reverse primers for *SlRBZ*. We cloned a 2.083-kb *SlRBZ* endogenous promoter fragment upstream of the start codon using the primers listed in Table S2. And the promoter *SlRBZ*:GUS fusion construct was generated by inserting the promoter fragment of *SlRBZ* in front of the GUS coding sequence. Transgenic plants carrying this construct were acquired as described earlier. The tissues of positive transformants were submerged in GUS staining solution overnight in the dark. Chlorophyll was then removed by incubation in 75% ethanol and the expression patterns were observed under a microscope.

### Subcellular localization of *SlRBZ* in tomato

To analyze the subcellular localization of SlRBZ, the full-length open reading frame (ORF) without the stop codon of *SlRBZ* was PCR-amplified using the primers containing *Kpn* I and *Bam*H I recognition sites (Table S2). The plasmids containing the correct sequence of *SlRBZ* were digested with *Kpn* I and *Bam*H I and introduced into pU1391 to create a fusion construct (pU1391-*SlRBZ*). The fusion construct and the control (pU1391) were bombarded into onion epidermal cells using the PDS-1000 system (Bio-Rad). The onion cells were cultured on MS medium and then observed with a confocal laser microscope (Leica TCSST2) 24 h after bombardment.

### RNA isolation and qRT-PCR analysis

Total RNA was extracted by TRIzol reagent (Invitrogen, USA). First-strand cDNA was synthesized using M-MLV reverse transcriptase (Toyobo, Japan) according to the supplier's protocols. The qRT-PCR was performed using the SYBR Green I Master Kit (Roche, Switzerland) on a LightCycler 480 Real-Time PCR Detection System, with β-actin transcripts as internal controls. The primers used for qRT-PCR analysis are listed in Table S1. The PCR amplification included a 30 s denaturation at 95°C, followed by 40 cycles of 95°C for 10 s, 58°C for 15 s, and 72°C for 20 s. Each cDNA sample was subjected to a qRT-PCR analysis in triplicate. The data were normalized using β-actin as the reference gene.

### Measurement of plant growth, Chl content, and net photosynthesis rate

Six randomly selected 1-month-old seedlings were collected to measure morphological indices, including plant height, leaf length, and leaf width. Chl content was measured following the procedure described by Wellburn (1994). First, leaf tissues were ground using liquid nitrogen. Chl was then extracted with 80% (v/v) acetone for an hour under low light intensity. Extraction was carried out several times during the reverse centrifugal tube to accelerate the process. Second, the samples were centrifuged at 12000 g for 10 min, and then the clear liquid was collected to determine Chl content. Finally, Chl content was determined by spectrophotometry. Net photosynthesis rate was measured by a gas exchange system of TPS-1 (PP Systems Company, UK). The experiments were performed thrice using independent biological replicates. The analysis of variance (ANOVA) with Bonferroni's post-test was conducted using SPSS.17.

### Carotenoid assay by HPLC

Carotenoids were extracted following the method described by Liu et al. with slight modifications (Liu et al., 2007). Carotenoids were determined on a reverse phase Analytical YMC Carotenoid Column C30 (150 × 4.6 mm i.d., 3 μm, Wilmington, NC, USA) using a Waters HPLC system with a photodiode array detector (Waters, Milford, MA). Operation was conducted under subdued light to avoid carotenoid degradation. Identification of carotenoids was performed by comparison with standard spectra. Quantification was performed using the calibration curve generated with commercially available lycopene, β-carotene, β-cryptoxanthin, lutein, and violaxanthin standards (Sigma-Aldrich).

### Quantification of endogenous GAs

Tomato leaf tissues were homogenized in liquid nitrogen and then the GA was extracted in 4 mL 80% (v/v) ice-cold aqueous methanol containing butylated hydroxytoluene (1 mmol/L) and polyvinylpyrrolidone (60 mg/g fresh weight). The samples were incubated overnight at 4°C and centrifuged at 10,000 g for 10 min. The resulting supernatants were individually collected and filtered through C18 Sep-Pak cartridges (Waters, Millford, MA, USA). The efflux was collected, and then dried in N_2_. Concentrations of GA_1+3_ and GA_4+7_ were measured by ELISA following methods described in previous publications (Zhu et al., 2005).

### Scanning electron microscope (SEM) observation

For SEM, the samples from transgenic and control seedlings were cut into small pieces of ~0.1 cm^3^ size, placed in 2.5% glutaraldehyde fluid, vacuum fixed for ~24 h, and then washed three times with 0.1 M phosphate buffer for 10 min. The samples were dehydrated in a series of ethanol, dried in HCP-2 (Hitachi), and coated with palladium–gold in an ion injection apparatus (JFC-1600). Observations were carried out on a scanning electron microscope (JEOL JEM-6390 LV).

### Transmission electron microscope (TEM) observation

The samples (cotyledons of 2-week-stage seedlings and true leaves from top to bottom of 6-week-stage seedlings) were fixed in 2.5% glutaraldehyde, vacuum fixed overnight, washed three times with 0.1 M phosphate buffer for 30 min, and then post-fixed with 1% osmiophilic tetroxide for 2 h. The fixed samples were dehydrated with a series of alcohol solutions, and then infiltrated and embedded in Spurr resin (SPI-812) with an acetone mixture. The ultra-thin sections were prepared with a Reichert Ultracut-6 (Leica Microsystems, Bannockburn, IL, USA), stained with uranyl acetate and lead citrate before TEM (Hitachi H-7650, Tokyo, Japan), and then photographed with Gatan 832 digital imaging system.

### RNA-sequencing and functional analysis of DEGs

Transgenic tomato plants overexpressing *SlRBZ* (OE-5) and wild-type AC were used for transcriptomic analysis. Total RNA was isolated from these materials, and the poly-A containing mRNA was purified from the total RNA using poly-T oligo-attached magnetic beads. The mRNA was fragmented into small pieces (250–350 bp) and then reversely transcribed into first-strand cDNA using random hexamers, followed by second-strand cDNA synthesis using DNA Polymerase I and RNase H. End repair of the double-stranded cDNA was performed to convert the overhangs into blunt ends and then purified. The libraries were constructed and sequenced using Illumina HiSeq® 2000. Clean reads were gained by removing the 3′ adaptor, low-quality reads, and reads less than 20 nt or containing two N. Gene expression levels were calculated using FPKM method (fragments per kilobase of transcript per million mapped reads). An absolute value of the log2 ratio ≥ 1 and *P* ≤ 0.05 were applied as thresholds to characterize the significance of gene expression level. The DEGs were annotated by aligning against the NCBI non-redundant nucleotide database. For further identification of pathways related to the *SlRBZ* gene, DEGs were compared with the Kyoto Encyclopedia of Genes and Genomes database (KEGG).

## Results

### Isolation and molecular characterization of *SlRBZ*

**Figure 1:**
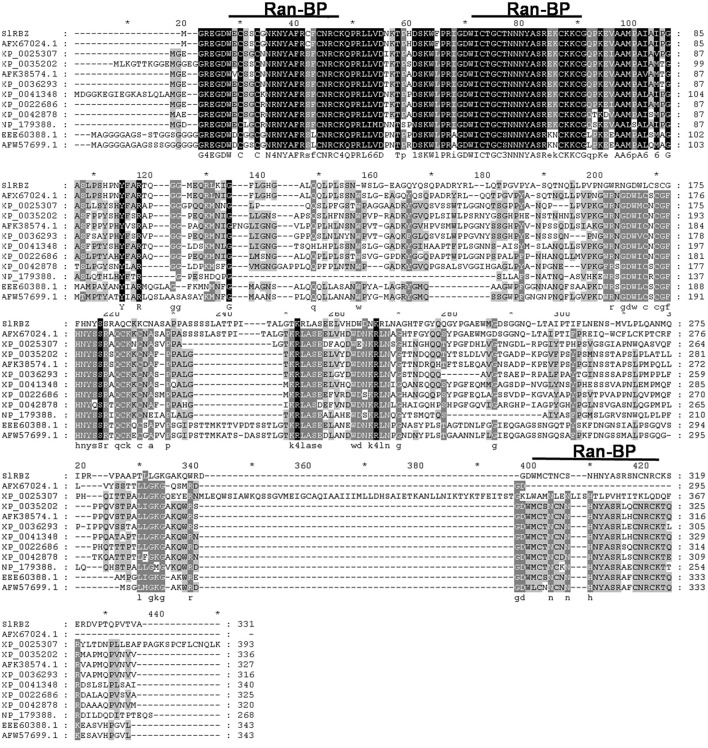
**Amino acid alignments of the protein encoded by ***SlRBZ*** with 11 homologs from other species**. Identical residues are highlighted in black, conserved residues in solid line.

We cloned and identified a Ran-binding protein zinc finger gene (named as *SlRBZ*) in tomato. Based on the nucleotide sequence of this gene (http://solgenomics.net/), the primers for the full length were designed. We isolated the full-length ORF from the tomato cultivar Ailsa Craig (AC) using RT-PCR. The predicted coding sequence of *SlRBZ* was 996 bp and encoded a protein of 331 amino acid residues. Through genomic and cDNA sequence alignment, we determined that *SlRBZ* contains seven exons and six introns. SlRBZ harbors three dispersed RanBP-type zinc fingers that conform to the RanBP2-type consensus sequence pattern (W-*X*C-*X*(2)-C-*X*(3)-N-*X*(6)-C-*X*(2)-C). Carrying out the pBLAST with SlRBZ amino acid sequence, the homologous proteins of other species were retrieved from the NCBI database. Alignments of SlRBZ with these homologous genes, including potato (AFX67024.1), ricinus (XP_0025307), soybean (XP_0035202), crowtoe (AFK38574.1), medicago (XP_0036293), cucumber (XP_0041348), *Vitis vinifera* (XP_0022686), strawberry (XP_0042878), *Arabidopsis* (NP_179388.), rice (EEE60388.1), and maize (AFW57699.1), indicated that all these genes contain three conserved domains (Figure 1). The amino acid sequence of SlRBZ has 90% sequence identity with the homolog in potato but only 51% sequence identity with the homolog in maize. The other homologs displayed ~51–90% sequence identity to SlRBZ.

### Subcellular localization and expression pattern of *SlRBZ*

**Figure 2:**
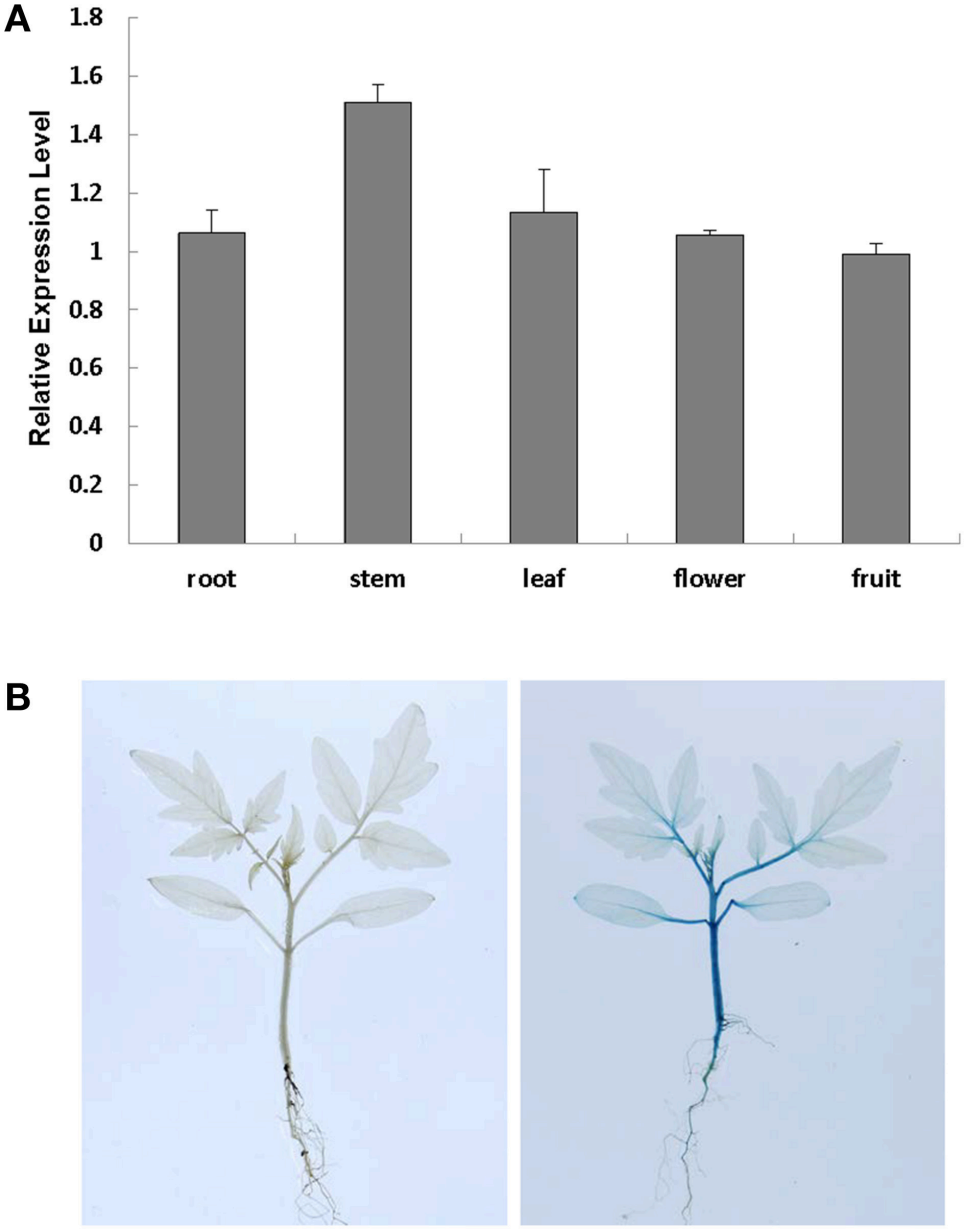
**Analysis of ***SlRBZ*** expression in tomato**. **(A)** Real-time RT-PCR analysis of *SlRBZ* expression in different tissues. **(B)** GUS expression in young seedlings, including roots, stems, leaves. Error bars indicate standard error (SE) of three replicates.

To study the subcellular localization of *SlRBZ*, we examined the GFP fluorescence in onion epidermal cells transformed with a fusion construct (pU1391-*SlRBZ-GFP*) and a control construct (pU1391-*GFP*). The fusion protein SlRBZ-GFP was localized in the nucleus, whereas GFPs alone were observed in the membrane and cytoplasm, manifesting that SlRBZ is a nuclear protein (Figure S1). To examine the expression pattern of S*lRBZ*, we carried out qRT-PCR analysis of the total RNA extracted from several tissues of AC, including roots, young stems, functional leaves, flowers, and green fruits. *SlRBZ* was expressed in various tissues, and the expression was much higher in the young stems (Figure 2A). We further carried out the transformation of *SlRBZ* promoter::GUS construct and analyzed the spatial expression pattern of *SlRBZ*. GUS was mainly detected in stems and petioles (Figure 2B).

### *SlRBZ* is induced by GA and IAA but suppressed by ABA

**Figure 3:**
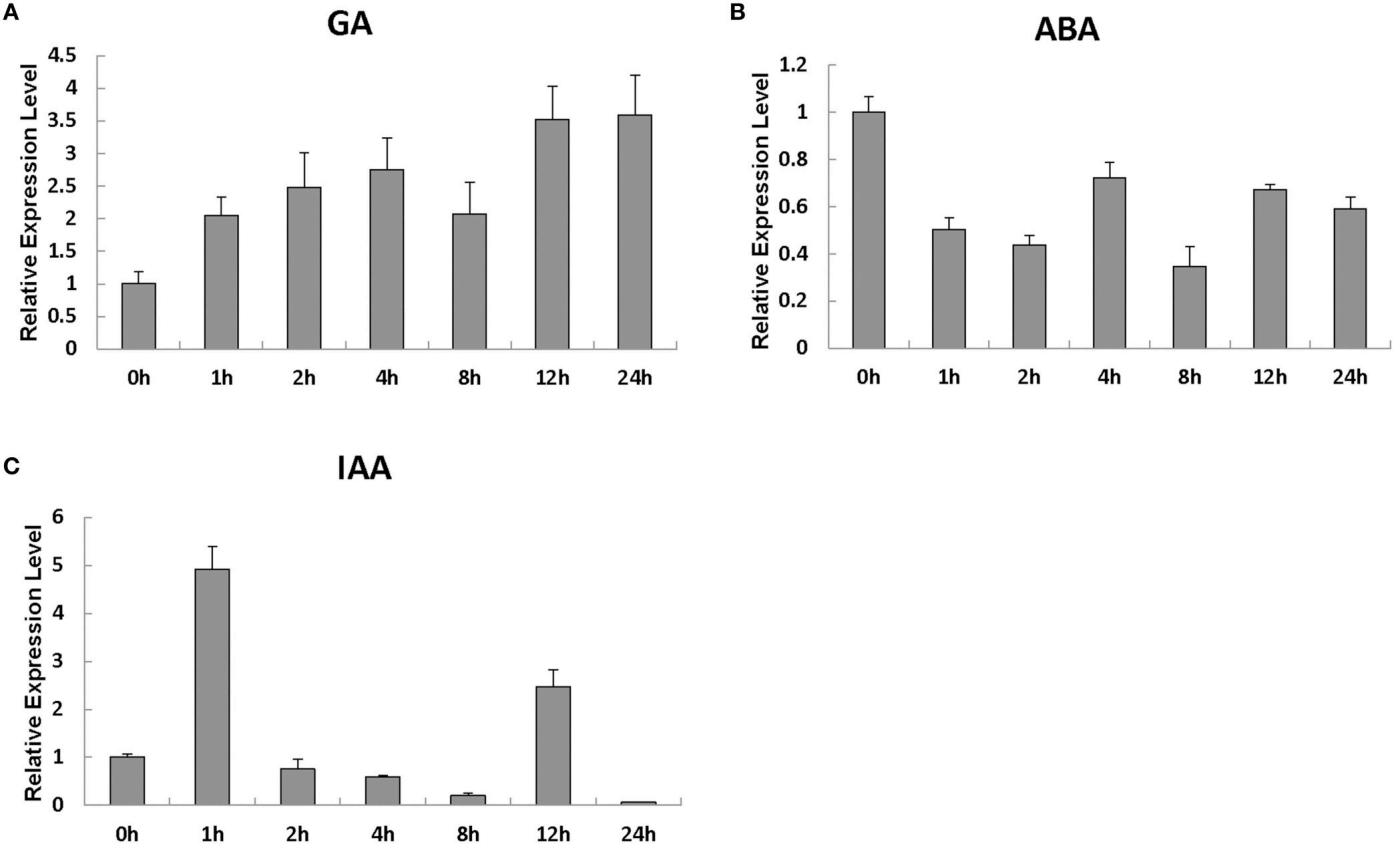
**Real-time RT-PCR analysis of the expression of ***SlRBZ*** in response to the phytohormones GA3 (A), ABA (B), and IAA (C)**. All samples were prepared at the indicated time points from three biological replicates in each group. Error bars indicate the SE of three replicates.

We evaluated the *SlRBZ* expression changes after treatment with different phytohormones. The investigation indicated that the transcript levels of *SlRBZ* substantially increased 1 h after GA treatment (Figure 3A). However, *SlRBZ* expression was evidently repressed from 1 to 24 h after ABA treatment (Figure 3B). In addition, we found that the transcription level of this gene reached a peak value 1 h after IAA treatment and then quickly returned to the lower levels 2 h later (Figure 3C). These results indicated that *SlRBZ* was positively regulated by GA and IAA, whereas negatively by ABA.

### Overexpression of *SlRBZ* caused chlorosis and dwarfism in tomato

**Figure 4:**
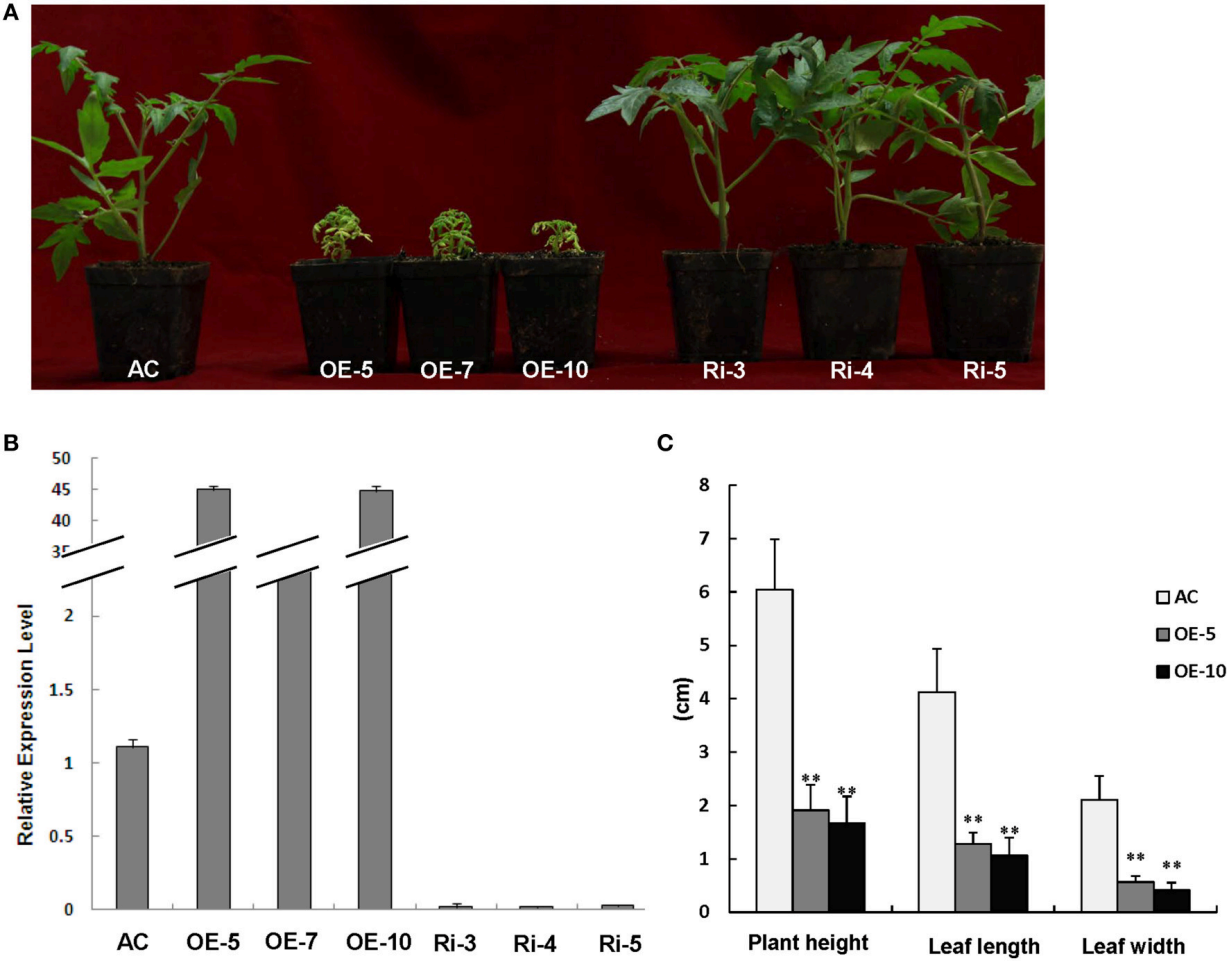
**Overexpression of ***SlRBZ*** results in chlorosis and dwarfism in tomato. (A)** Phenotypes of seedlings of *SlRBZ*-overexpressing tomato OE-5, OE-7, and OE10, and RNAi-inhibiting expression lines Ri-3, Ri-4, and Ri-5, and AC plants. **(B)** The expression level of *SlRBZ* in transgenic and AC plants. **(C)** Phenotype analysis **(C)** in wild type AC and *SlRBZ-OE* transgenic tomato plants. Error bars indicates SE (*n* = 3). Asterisks indicate significant differences compared with WT (^**^P < 0.01).

We characterized the function of *SlRBZ* by generating transgenic tomato plants with overexpression or RNAi silencing of *SlRBZ*. 12 *SlRBZ* overexpression (OE) and 17 RNAi knock-down (Ri) tomato lines were obtained, respectively. The transgenic plants and AC plants grew under the same conditions. And the overexpression plants exhibited a severely etiolated and dwarf phenotype, whereas no obvious change of morphology was observed in *SlRBZ*-Ri lines (Figure 4A). The expression level of the *SlRBZ* gene in transgenic and AC plants was examined by qRT-PCR. The expression of *SlRBZ* was evidently upregulated in *SlRBZ*-OE lines and downregulated in *SlRBZ*-Ri lines (Figure 4B). Therefore, we selected the overexpression transgenic lines and AC for further studies. We measured plant height, leaf length and width of *SlRBZ*-OE plants. The average plant height of OE-5 and OE-10 plants was 1.91 and 1.67 cm, respectively, which was considerably lower than that of AC (6.04 cm; Figure 4C). The leaves of the *SlRBZ*-overexpression plants were considerably smaller than those of AC. The mean leaflet length of AC, OE-5, and OE-10 lines was 4.12, 1.28, and 1.06 cm, respectively, suggesting that OE-5 and OE-10 plants were 31.07 and 25.73% smaller than AC, respectively (Figure 4C). In addition, the mean leaflet width was considerably smaller in transgenic plants compared with controls (Figure 4C). To investigate whether the cell size was influenced in *SlRBZ-OE* plants, the cell number per unit area in the upper epidermis were observed by SEM. The average cell number was 10 in each field of view (1500 × : ≈0.0015 mm^2^) in AC, whereas that was 25 in *SlRBZ-OE* plants (Figure S2). We thus, concluded that the cell size was depressed in SlRBZ-OE plants.

### Chl synthesis and photosynthesis were inhibited in *SlRBZ* overexpressing plants

**Figure 5:**
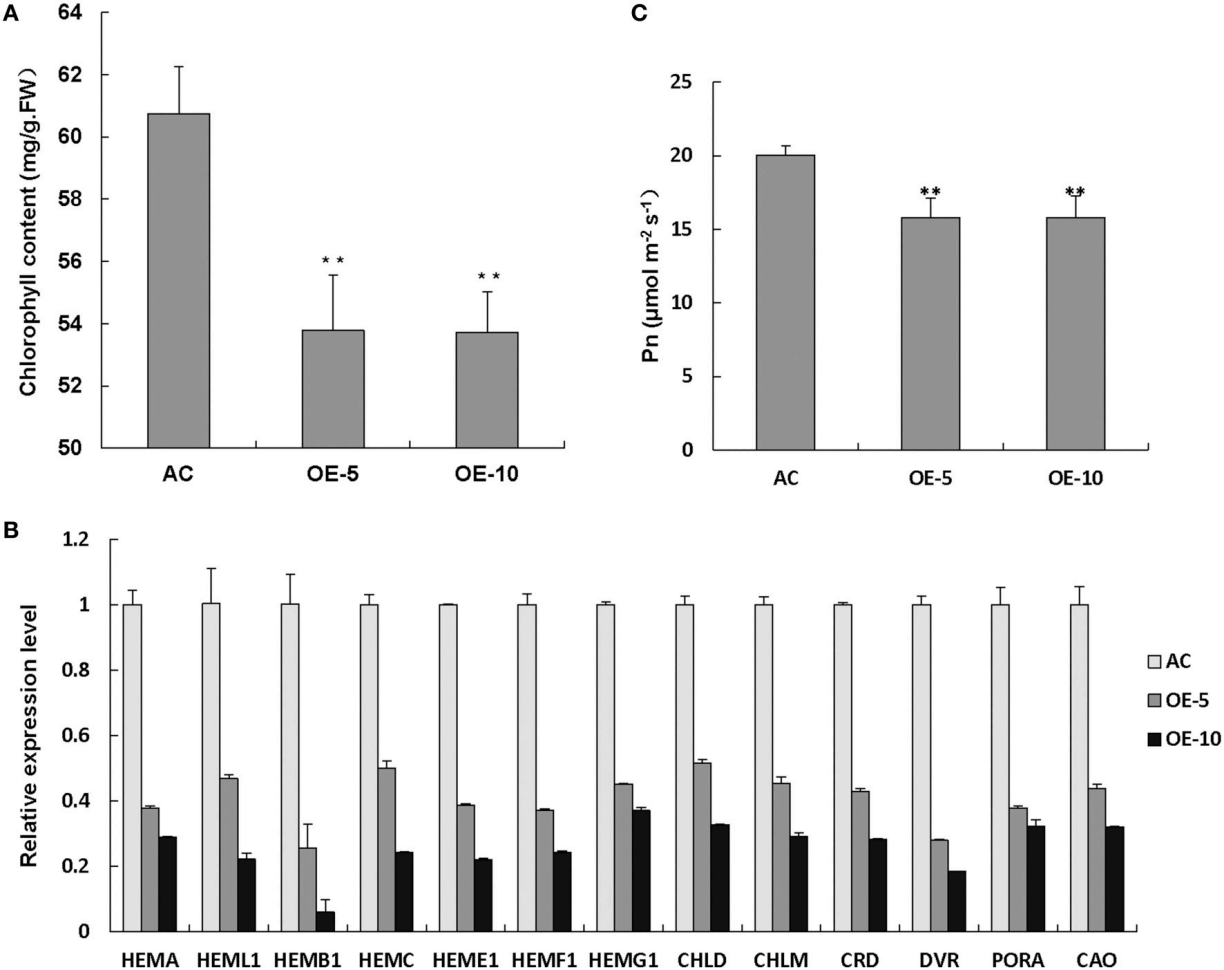
**Chl synthesis and photosynthesis are inhibited in ***SlRBZ*** overexpressing plants**. Chlorophyll content **(A)**, expression levels of chlorophyll biosynthetic genes **(B)** and leaf net CO_2_ assimilation rate **(C)** in wild type AC and *SlRBZ*-OE transgenic tomato plants. Error bars indicates SE (*n* = 3). Asterisks indicate significant differences compared with WT (^**^P < 0.01).

To determine the reasons for leaf chlorosis, we measured the Chl content and found that Chl levels were apparently reduced in *SlRBZ* overexpressing plants compared with the wild-type AC (Figure 5A). In addition, expression of Chl biosynthetic genes, including *HEMA, HEML1, HEMB1, HEMC, HEME1, HEMF1, HEMG1, CHLD, CHLM, CRD, DVR, PORA*, and *CAO*, was compared between *SlRBZ* transgenic plants and AC seedlings, and all genes in the Chl biosynthetic tetrapyrrole pathway were downregulated in *SlRBZ* transgenic plants (Figure 5B). To establish whether or not *SlRBZ* impacts photosynthetic capacity, leaf net CO_2_ assimilation rate (Pn) was measured by a gas exchange system of TPS-1. Results showed that the Pn of overexpressed *SlRBZ* plants was inhibited in comparison with AC (Figure 5C).

### Carotenoid contents and transcription level of genes related to carotenoid biosynthesis were decreased in *SlRBZ*-OE plants

**Figure 6:**
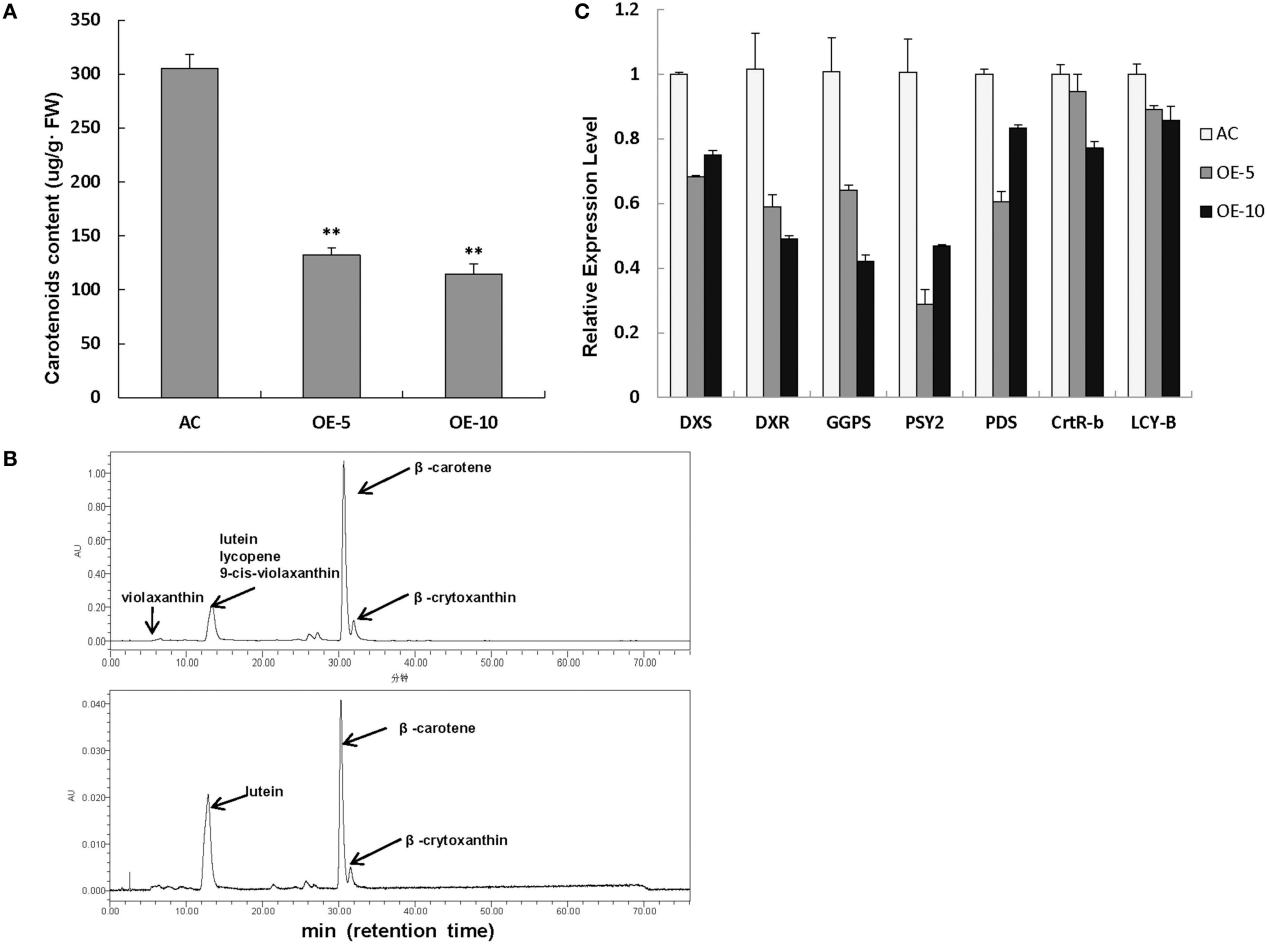
**Carotenoid contents are decreased in SlRBZ-OE plants. (A)** Carotenoids content were measured by spectrophotometer. **(B)** Carotenoids content were measured by HPLC (Upper: wild type AC plants; Lower: *SlRBZ*-OE transgenic tomato plants OE-5). **(C)** Expression levels of carotenoids biosynthetic genes and expression levels of genes involved in the early steps of carotenoid, chlorophyll, and GA biosynthesis pathways. Error bars indicates SE (*n* = 3). Asterisks indicate significant differences compared with WT (^**^P < 0.01).

As we know, both Chl and carotenoids are synthesized in plant leaves and contribute to the leaf color. Therefore, the total carotenoid content was also measured by the spectrophotometer. The level of carotenoids was markedly decreased in *SlRBZ* transgenic plants in comparison with AC (Figure 6A). For details, several compounds of carotenoids, such as lutein, β-carotene, and β-crytoxanthin, was further detected by HPLC. Lutein, β-carotene, and β-crytoxanthin contents were 71.83, 289.46, and 22.60 μg/g·FW, respectively, in AC leaves (Figure 6B). By contrast, these compounds were 6.54, 28.94, and 3.66 μg/g·FW, respectively, in *SlRBZ* overexpressing plants (Figure 6B). In other words, the content of lutein, β-carotene, and β-crytoxanthin was 9.1, 10, and 16.2% lower than AC. In addition, violaxanthin, lycopene, and 9-cis-violaxanthin could be detected in AC, whereas these compounds were absent in *SlRBZ* overexpressing plants (Figure 6B). Furthermore, we examined the transcriptional changes of genes correlated with carotenoid biosynthesis, including *PSY2, PDS, CRTR-B1*, and *LYC-B*. Results showed that the expression levels of these genes evidently decreased in *SlRBZ* overexpressing plants (Figure 6C). Furthermore, several genes involved in the early steps of Chl, carotenoid, and GA biosynthesis, like *DXS, DXR*, and *GGPS*, were also considerably lower in the *SlRBZ* overexpressing plants than that in AC (Figure 6C).

### *SlRBZ* overexpressing plants exhibit a classical reduced GA phenotype

**Figure 7:**
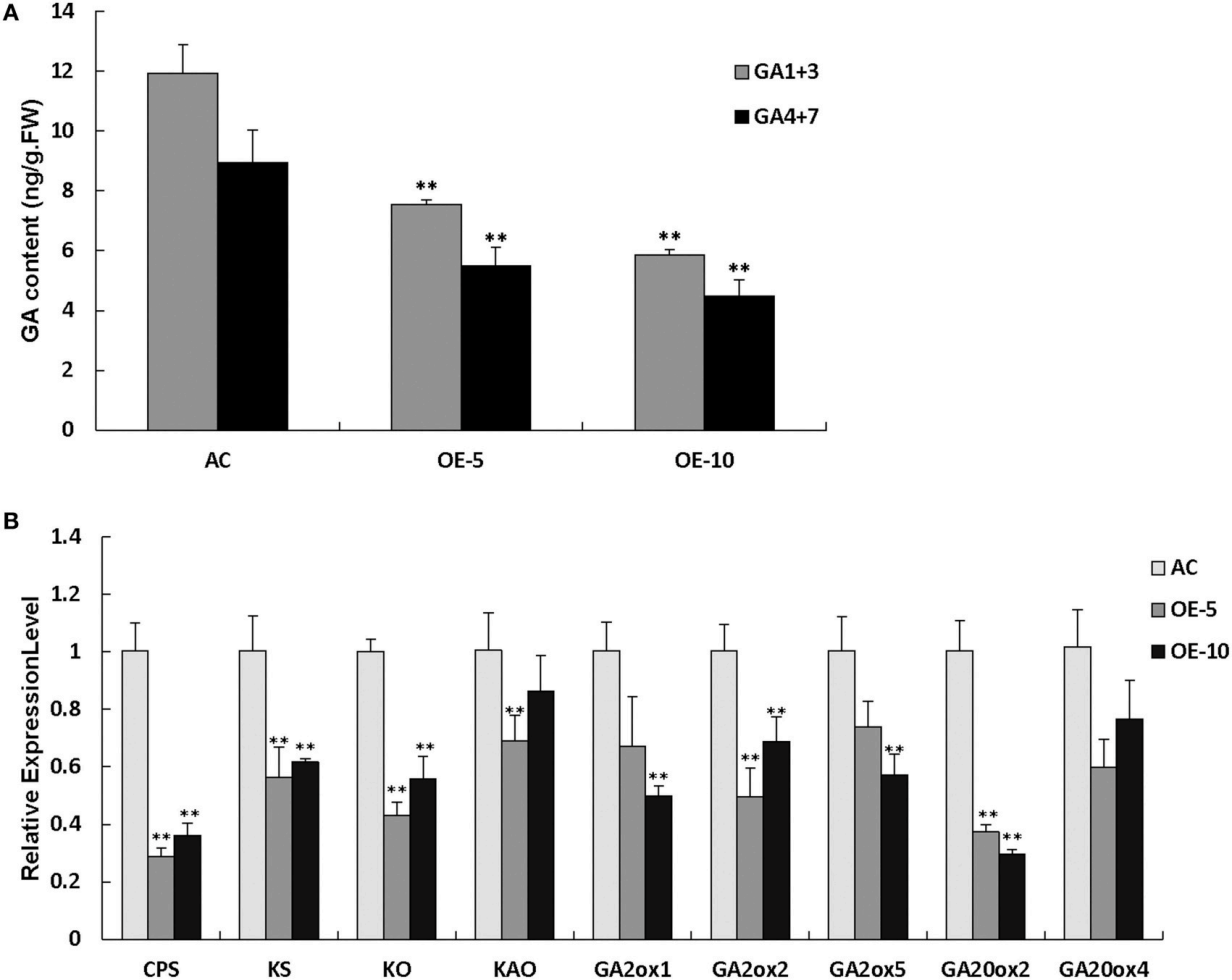
**GA_**1+3**_ and GA_**4+7**_ are evidently decreased in transgenic plants compared with AC. (A)** GA_1+3_ and GA_4+7_ content were measured by an indirect ELISA method with anti-GA antibodies. **(B)** qRT-PCR analysis of GAs biosynthetic genes. Error bars indicate the SE of three replicates. Asterisks indicate significant differences compared with WT (^**^P < 0.01).

The *SlRBZ* overexpressing transgenic plants showed dwarf phenotypes. We thus, measured the content of endogenous GAs. Results indicated that the two types of GAs (GA_1+3_ and GA_4+7_) were evidently decreased in transgenic plants compared with AC (Figure 7A). To further determine whether or not the synthesis of GA was influenced by *SlRBZ*, we compared the expression of GA biosynthetic genes between *SlRBZ* transgenic and AC plants. Previous studies indicated that *KO, KAO, CPS*, and *KS* have important functions in the early steps of GA biosynthesis. The qRT-PCR analysis showed that the expression of these genes was dramatically downregulated in *SlRBZ* transgenic plants (Figure 7B). In addition, the transcriptional levels of five genes in the GA biosynthetic pathway, namely, *SlGA2ox1, SlGA2ox2, SlGA2ox5, SlGA20ox2*, and *SlGA20ox4*, which determine GA concentration in many plants, were also repressed in transgenic plants (Figure 7B). Furthermore, spraying with 100 μM GA_3_ on *SlRBZ* overexpressing plants every 3 d can significantly rescue the plant height back to control levels, demonstrating that *SlRBZ* overexpressing plants exhibit a classical reduced GA phenotype (Figure S3).

### Overexpressing *SlRBZ* can affect the chloroplast development in tomato

**Figure 8:**
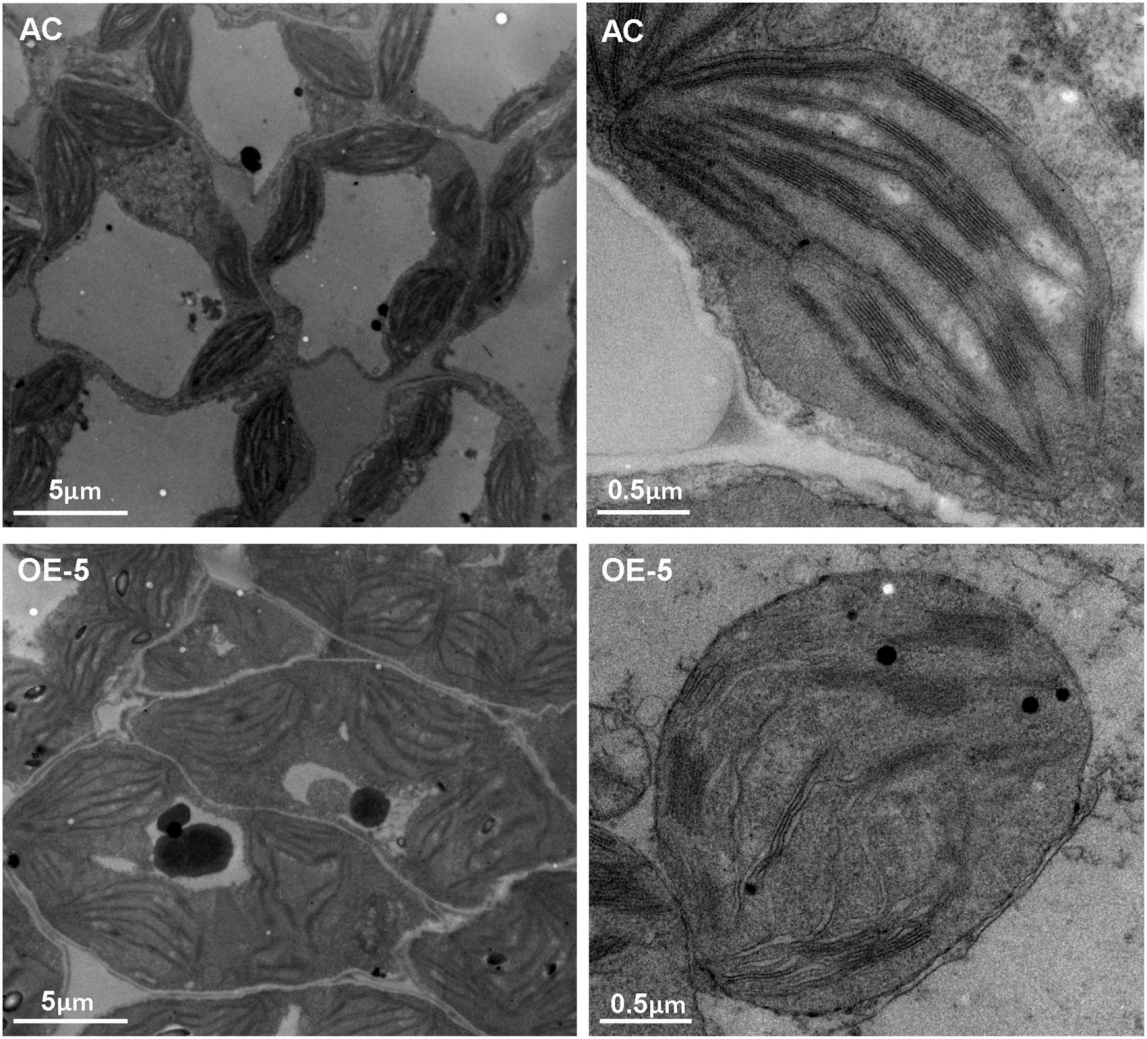
**Transmission electron microscopy of chlopolasts in wild type AC and ***SlRBZ***-OE transgenic tomato plants (OE-5)**.

Overexpression of *SlRBZ* resulted in leaf chlorosis and reduced total Chl content. Moreover, chloroplast development was closely related with Chl content. We thus, observed the chloroplast ultrastructure by TEM in the transgenic tomato leaves. Observations indicated that chloroplasts in AC leaves were normal, including the thylakoid membrane system, lamellar layer system of the thylakoid, and the inner and outer membrane system (Figure 8). However, the ultrastructure of chloroplasts in *SlRBZ* overexpressing plants was evidently blocked. For example, the membranes of chloroplast and grana and stroma thylakoid exhibited different degrees of rupture and collapse; grana lamellae were significantly less than AC; the stromata were disintegrated (Figure 8). In addition, the thylakoid was cracked, the number and volume of osmiophilic globules increased and the configurations of the thylakoid systems nearly disappeared (Figure 8). Furthermore, the number of granum-stroma thylakoid membranes in overexpressed *SlRBZ* plants significantly decreased (Figure 8). Taken together, these results demonstrated that *SlRBZ* has a critical function in chloroplast formation.

### *SlRBZ* inhibits the expression of Chl and photosynthesis-related genes

To further characterize the molecular mechanism that triggers the phenotype of chlorosis and dwarfism mediated by *SlRBZ*, we further compared the gene expression profiles of *SlRBZ* transgenic and AC plants by RNA-Seq analysis (The transcriptome data about the transgenic tomato plants SlRBZ-OE and wild-type AC are available at http://www.ncbi.nlm.nih.gov/geo/query/acc.cgi?acc = GSE77340). Clean reads were obtained by discarding low-quality reads, resulting in 22,824,878 clean reads for AC and 22,958,774 clean reads for *SlRBZ*-OE. Among them, 13,836,447 (60.62%) clean reads for AC, and 12836008 (55.91%) clean reads for *SlRBZ*-OE were mapped to the reference genome ITAG2.4, suggesting that the RNA-seq transcriptomes were sufficient for subsequent gene expression analyses. The putative differentially expressed genes (DEGs) between *SlRBZ*-OE and AC plants were identified by applying fold changes (*FC* ≥ 2 or *FC* ≤ 0.5), and (*P* ≤ 0.01) were applied as standards to determine the significance levels of DEGs. On the basis of these criteria, 515 genes were identified to be upregulated in the *SlRBZ*-OE plants compared with AC. Meanwhile, the transcription of 568 genes was decreased by more than twofold in the *SlRBZ*-OE plants. For biological explanation of these DEGs, we analyzed these genes using the KEGG pathway. Results indicated that overexpression of *SlRBZ* can repress the expression of genes involved in Chl synthesis pathway, including *por, hemE*, and *chlM* (Table S1). Furthermore, the expression levels of photosynthetic genes, such as *Psa E, Psa F, Psa H* (Photosystem I), *Psb P, Psb Q, Psb W, Psb 27* (Photosystem II), and *Pet F* (photosynthetic electron transport), were evidently repressed in the *SlRBZ*-OE plants (Table S1). In addition, in accordance to our previous results, some GA biosynthesis-related genes were also detected and showed significantly lower expression levels in *SlRBZ*-OE plants than in Wt, such as GA20ox-2 (Solyc06g035530) and GA2ox-1 (Solyc05g053340; Figure 7B).

### *SlRBZ* inhibits the expression of many light-harvesting Chl a/b-binding protein genes (Lhca/b) and chloroplast protein genes

The *Lhcb* genes encoding membrane proteins, which consist of the antennae complexes and capture and transfer light energy to the reaction centers of photosystem I and photosystem II (Armond et al., 1977), are abundant in plants. The transcriptional levels of the *Lhcb* genes are induced by many developmental cues and environmental signals, such as chloroplast formation, light, and oxidative stress (McCormac and Terry, 2002; Staneloni et al., 2008). Therefore, their expression is severely repressed in tissues without mature chloroplasts. In accordance to this conclusion, we found that the expression of many *Lhc*a/*b* genes were significantly downregulated in *SlRBZ*-OE plants, including Solyc03g005790, Solyc06g069730, Solyc04g082920, Solyc04g082930, Solyc12g011280, Solyc12g009200, Solyc08g067320, Solyc08g067330, CAB and Solyc08g007180 (Table S1). As *Lhcb* genes are reporters of chloroplast formation, we speculate that chloroplasts must be blocked in *SlRBZ*-OE plants. In addition, thylakoid lumenal proteins are necessary for the formation of photosystem II complexes (Hou et al., 2015). The expression of genes encoding thylakoid lumenal proteins must be repressed without normal photosystem II complexes in etiolated plants. Consistent with the foregoing, many genes encoding thylakoid lumenal proteins were also downregulated in transgenic plants, such as Solyc01g087040, Solyc03g082890, Solyc06g066620, and Solyc10g084040 (Table S1).

## Discussion

ZFPs are important regulators and widespread in nature. They participate in multiple biological processes, including development, plant architecture, phytohormone response, and stress response (Laity et al., 2001; Li et al., 2013). Ran-binding proteins are also characterized as ZFPs that contain two conserved Ran-BP domains. This type of ZFPs was first discovered in the nuclear export protein RanBP2 (Gamsjaeger et al., 2007). Its functions have been studied in humans and animals (Higa et al., 2007). However, it remains largely uncharacterized in plants. The vast knowledge on ZFPs in animals benefits their discoveries in plants. Only a RanBP2 zinc finger protein gene was identified in upland cotton, which is expressed in the different development stages of glands (Chang et al., 2007). Similar to other ZFPs, *SlRBZ* is highly conserved in different species. In this study, we found that *SlRBZ* acts as a novel regulator controlling the biosynthesis of Chl, carotenoid, and GA in tomato. As previously reported, *WRKY53* and *OsDOS* participate in the control of leaf senescence, which belongs to other subfamilies of ZFPs (Miao et al., 2004; Kong et al., 2006). Thus, we infer that *SlRBZ* may influence Chl synthesis in plants through the common mechanisms with other ZFPs. In addition, the expression level of *SlRBZ* is significantly higher in tomato plants treated with exogenous GA than in controls, suggesting that this gene is positively controlled by GA.

*SlRBZ* encodes a RanBP transcription factor that is localized in the nucleus. Overexpression of *SlRBZ* results in etiolated and dwarf phenotype in tomato. Notably, this phenotype appears at the early developmental stage. qRT-PCR analyses indicate that *SlRBZ* is expressed in almost all tissues. Chl, carotenoids, and GAs are synthesized in chloroplasts (Xing et al., 2010). We measured the contents of Chl, carotenoid and GAs in the *SlRBZ-*OE transgenic plants, and confirmed that these three chemical substances were evidently decreased. Moreover, the expression of the genes involved in their biosynthesis pathways were also inhibited in *SlRBZ* overexpressing plants, such as *por, hemE, chlM, PSY2, PDS, ZDS, CRTR-B1, LYC-B, KO, KAO, CPS*, and *KS*, suggesting that chloroplast development must be repaired.

Ultrastructure observation showed that chloroplasts are blocked in *SlRBZ* overexpressing plants, particularly the membranes of chloroplast and grana, and thylakoid stroma appeared to be ruptured and collapsed. Previous studies have demonstrated that mutations in *pds3* and *zds* mutants block chloroplast development (Dong et al., 2007; Qin et al., 2007). HCF164, a thioredoxin-like protein, also participates in chloroplast development, and its mutation causes abnormal chloroplast morphology (Lennartz et al., 2001). The expression of its homologs (Solyc07g064940, Solyc08g006720, Solyc02g087850, Solyc01g087520, Solyc09g074570, etc.) was downregulated in *SlRBZ-*OE tomato plants. In addition, many thylakoid lumenal proteins were evidently affected in *SlRBZ-*OE tomato plants, which were encoded by the nuclear genes and localized in chloroplasts. Koussevitzky et al. (2007) showed that abnormal chloroplasts influenced the expression of plastid-localized genes (Koussevitzky et al., 2007). Furthermore, transcriptome analyses indicated that many genes correlated with photosynthesis and chloroplast differentiation are repressed in *SlRBZ-*OE plants. More importantly, the Lhcbs are used as the reporter genes for chloroplast development, and their expression are severely repressed in plants without mature chloroplasts (Larkin et al., 2003). In accordance with this result, numerous Lhcbs were significantly downregulated in *SlRBZ-*OE plants. From these results, we infer that the chlorosis and dwarfism phenotype in *SlRBZ-*OE tomato plants result from the impaired chloroplasts and then the reduction of Chl, carotenoid, and GAs synthesis.

Chloroplasts are essential for photosynthesis, and then plant vitality and growth (Pogson and Albrecht, 2011). The formation of the chloroplasts is codetermined by the nuclear- and plastid-encoded genes, such as *SLP, APG2*, and *PAC* (Taylor, 1989). However, to date, very few transcription factors have been identified to control chloroplast formation. *Golden2-like* genes, which belong to the GARP family of transcription factors, are required for chloroplast development (Waters et al., 2008). In addition, the GATA transcription factors (*GNC* and *CGA1*) also participate in chloroplast development in *Arabidopsis* (Chiang et al., 2012). In this study, we determined that the overexpression of *SlRBZ* can block chloroplast formation and the membranes of chloroplast and grana and stroma thylakoid in *SlRBZ* overexpressing plants ruptured and collapsed, demonstrating that *SlRBZ* regulates the formation of chloroplast in tomato. Similarly, *CYO1*, which encodes a protein disulfide isomerase, specifically regulates chloroplast biogenesis in *Arabidopsis* (Shimada et al., 2007). The number of grana thylakoids is closely related to Chl content (Anderson et al., 1973). Moreover, the genes relevant to chloroplastid proteins can reduce the stack of thylakoid and result in pale plants and etiolated phenotype (Jarvis et al., 1998; Huang et al., 2009; Barry et al., 2012). Consistent with this conclusion, our results showed that the Chl content was evidently decreased in *SlRBZ*-OE transgenic plants. In a previous report, the *Lhc* gene family was repressed in tissues lacking mature chloroplasts (Larkin et al., 2003). In accordance with this result, *Lhcb* genes as reporters of the formation of chloroplast, were repressed in *SlRBZ*-OE plants. In the present study, we found that net photosynthetic rate in *SlRBZ*-OE transgenic plants was just 78.91% of the Wt rate. Photosynthetic rate was repressed if grana lamella was reduced (Osborne and Raven, 1986). In general, Chl, carotenoid, and gibberellin biosyntheses were impaired in chloroplast, which is consistent with the reduced expression of most of genes involved in the tetrapyrrole biosynthetic pathway, as well as MEP and GA pathways in *SlRBZ*-OE transgenic plants (Figure S4). Therefore, we speculate that overexpression of *SlRBZ* may inhibit Chl, carotenoid, and GA biosynthesis by destroying chloroplast structure resulting in chlorosis and dwarfism in transgenic tomato plants. In accordance with this conclusion, depression of *PDS, DXR*, and *HST* participating in MEP, the PQ pathway, and carotenoid biosynthesis resulted in albino and dwarf phenotypes in *Arabidopsis* (Qin et al., 2007; Xing et al., 2010; Chao et al., 2014). These conclusions provide new insights the role of this novel RanBP2 zinc finger protein in tomato Chl, carotenoid, and GA biosynthesis. Future studies on this gene using ChIP-seq will provide more information underlying its regulatory mechanisms.

## Author contributions

ZY designed research; MF, SG, JR, and QY performed research; MF and CY analyzed data and wrote the paper.

### Conflict of interest statement

The authors declare that the research was conducted in the absence of any commercial or financial relationships that could be construed as a potential conflict of interest.

## Supplementary material

Figure S1 **Subcellular localization of SlRBZ**. Constructs of GFP and SlRBZ-GFP were transiently expressed in onion epidermal cells. GFP was localized in the entire onion cells **(A-C)**, SlRBZ -GFP was localized in nucleus **(D-F)**. Bright-field images **(A,D)**, GFP fluorescent images **(B,E)**, and merged images **(C,F)**.

Figure S2 **(A)** Cell morphology of leaf epidermis in AC and OE-5 plants. **(B)** Cell number was counted in each field of view (1500 × : ≈0.0015mm^2^) on at least 20 microscopes from three plants. Asterisks indicate significant differences compared with WT (^**^P < 0.01).

Figure S3 **The dwarf phenotype of SlRBZ-OE plants was rescued by spraying with GA3**. A Growth response of treated with 100 μM GA3 on AC and SlRBZ-OE plants. b Plant height was measured before and after spraying GAs.

Figure S4 **The expression of genes participating in carotenoid, chlorophyll and GA biosynthesis pathways were obviously decreased in SlRBZ-OE plants**.

Table S1 **Expression of genes involved in photosynthesis and chlorophyll biosynthesis in the SlRBZ-OE plants**.

Table S2 **Primers sequences used for functional and expression analysis in this study**.
