# Supplementary figures and images for: Overexpression of SlRBZ Results in Chlorosis and Dwarfism through Impairing Chlorophyll, Carotenoid, and Gibberellin Biosynthesis in Tomato
Source: Front Plant Sci. 2016 Jun 22;7:907. doi: 10.3389/fpls.2016.00907 (PMC4916219; doi:10.3389/fpls.2016.00907)

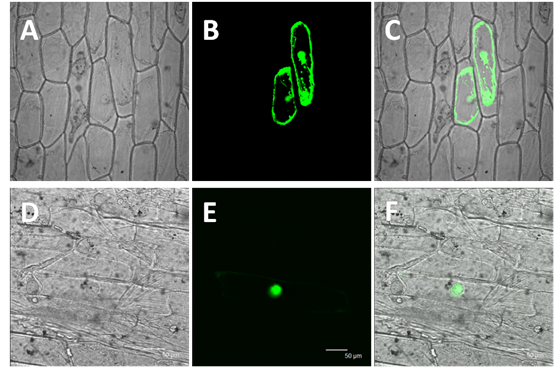

Supplement: Figure S1 — Subcellular localization of SlRBZ. Constructs of GFP and SlRBZ-GFP were transiently expressed in onion epidermal cells. GFP was localized in the entire onion cells (A-C), SlRBZ -GFP was localized in nucleus (D-F). Bright-field images (A,D), GFP fluorescent images (B,E), and merged images (C,F). [file Image1.TIFF]

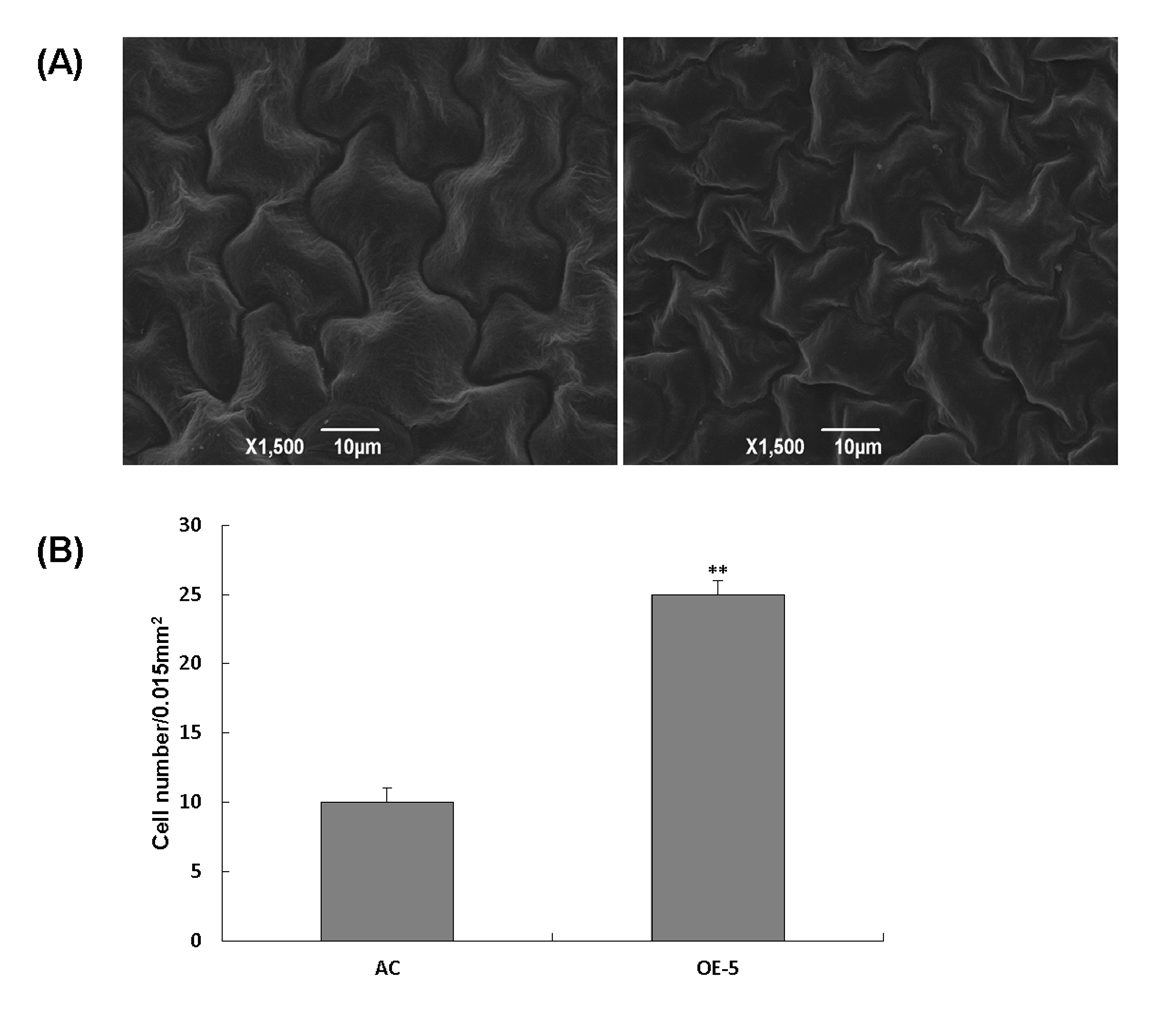

Supplement: Figure S2 — (A) Cell morphology of leaf epidermis in AC and OE-5 plants. (B) Cell number was counted in each field of view (1500 × : ≈0.0015mm2) on at least 20 microscopes from three plants. Asterisks indicate significant differences compared with WT (**P < 0.01). [file Image2.TIFF]

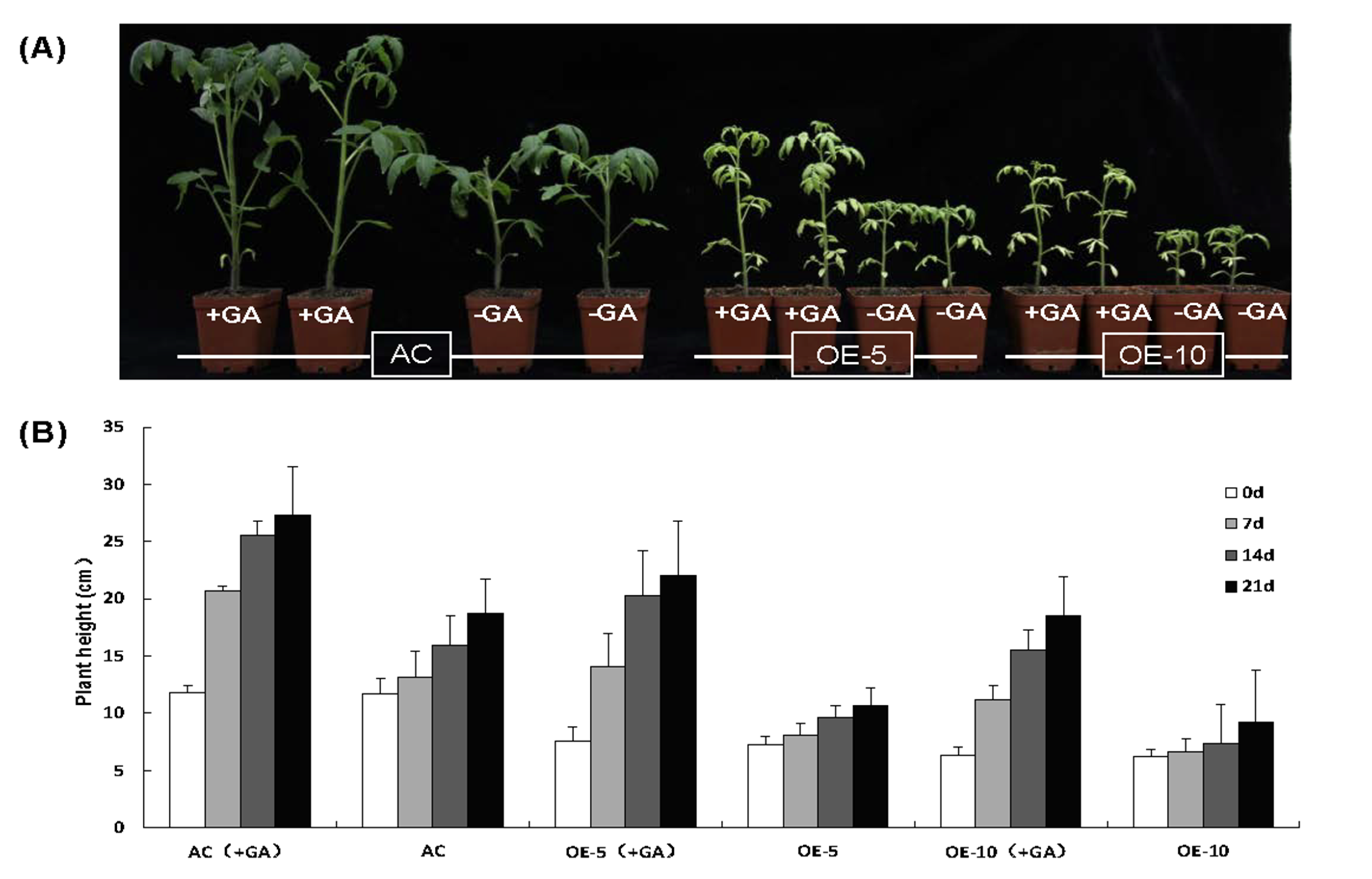

Supplement: Figure S3 — The dwarf phenotype of SlRBZ-OE plants was rescued by spraying with GA3. A Growth response of treated with 100 μM GA3 on AC and SlRBZ-OE plants. b Plant height was measured before and after spraying GAs. [file Image3.TIFF]

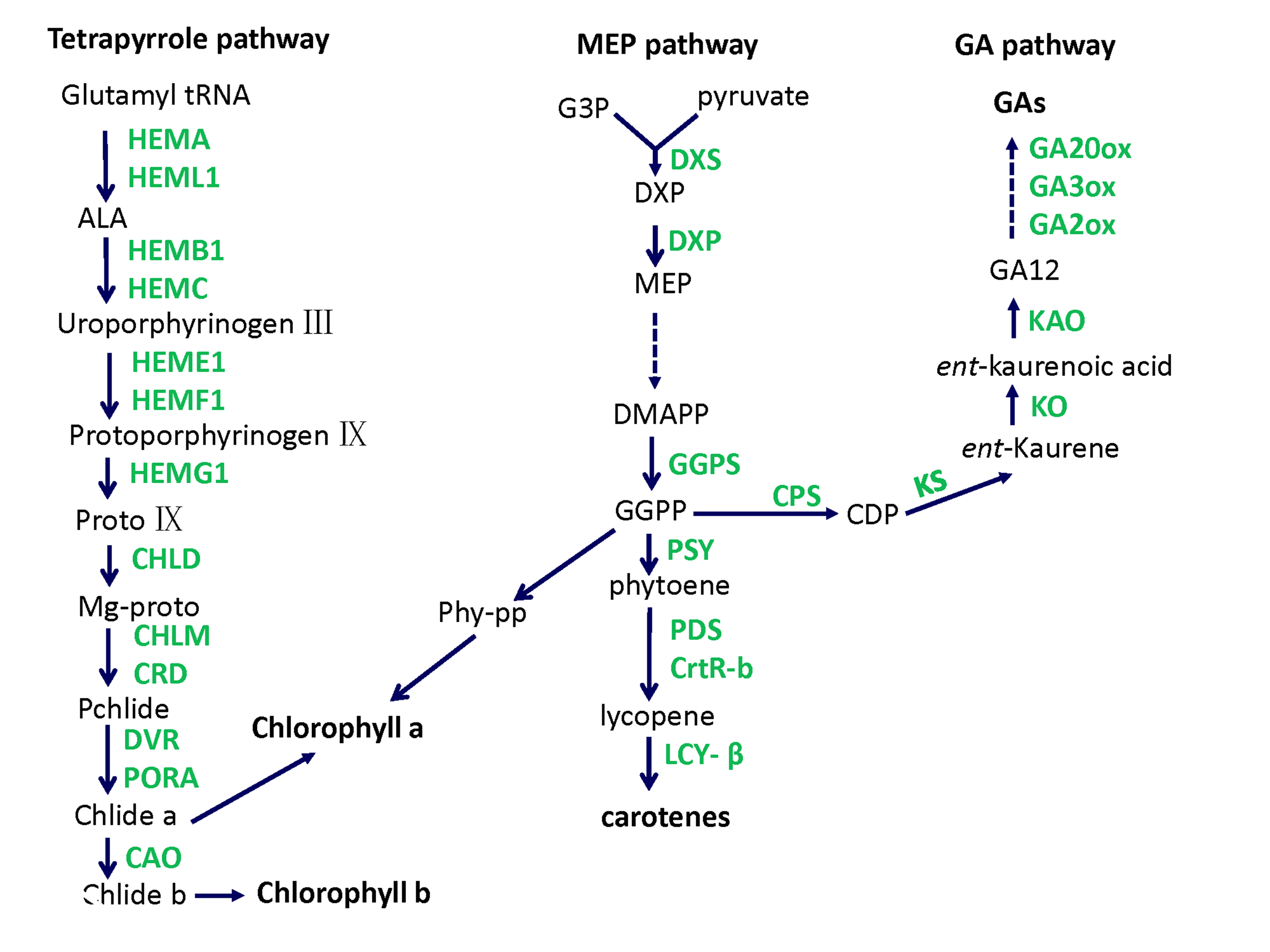

Supplement: Figure S4 — The expression of genes participating in carotenoid, chlorophyll and GA biosynthesis pathways were obviously decreased in SlRBZ-OE plants. [file Image4.TIFF]
