# Supplementary material for: Overexpression of SlRBZ Results in Chlorosis and Dwarfism through Impairing Chlorophyll, Carotenoid, and Gibberellin Biosynthesis in Tomato
Source: Front Plant Sci. 2016 Jun 22;7:907. doi: 10.3389/fpls.2016.00907 (PMC4916219; doi:10.3389/fpls.2016.00907)
Supplement: Table S1 — Expression of genes involved in photosynthesis and chlorophyll biosynthesis in the SlRBZ-OE plants. [file Table1.DOCX]

**Table S1**. Expression of genes involved in photosynthesis and chlorophyll biosynthesis in the SlRBZ-OE (OE-5) plants.

| Gene | Expression(FPKM) | | Expression(FPKM) | | | Descriptions |  |  |  |  |  |  |  |
| --- | --- | --- | --- | --- | --- | --- | --- | --- | --- | --- | --- | --- | --- |
|  | OE-5 | AC | log2(fold_change) | P-Value | Q-Value |  |  |  |  |  |  |  |  |
| Solyc12g013710.1 | 19.0 | 782.5 | -5.4 | 1.00E-05 | 0.0125416 | Protochlorophyllide reductase, POR1 |  |  |  |  |  |  |  |
| Solyc10g006900.2 | 30.1 | 521.2 | -4.1 | 0.000249939 | 0.0988108 | Protochlorophyllide reductase, POR1 |  |  |  |  |  |  |  |
| Solyc06g069730.2 | 154.1 | 2651.1 | -4.1 | 0.00225627 | 0.267121 | Chlorophyll a-b binding protein 4, LHCA4 |  |  |  |  |  |  |  |
| Solyc02g067360.2 | 3.7 | 60.8 | -4.0 | 0.00355582 | 0.333162 | Protease Do-like 8, DEGP8 |  |  |  |  |  |  |  |
| Solyc10g086390.1 | 3.7 | 58.5 | -4.0 | 0.00414871 | 0.352913 | Short-chain dehydrogenase TIC 32, TIC32 |  |  |  |  |  |  |  |
| Solyc11g005340.1 | 11.2 | 153.6 | -3.8 | 0.000394876 | 0.124151 | Probable plastid-lipid-associated protein 7, PAP7 |  |  |  |  |  |  |  |
| Solyc03g005790.2 | 32.4 | 424.4 | -3.7 | 1.47E-05 | 0.0155202 | Chlorophyll a-b binding protein 1D ，CAB1D |  |  |  |  |  |  |  |
| Solyc12g094720.1 | 27.7 | 361.7 | -3.7 | 0.00332644 | 0.325859 | PsbP domain-containing protein 3, PPD3 |  |  |  |  |  |  |  |
| Solyc12g011280.1 | 124.5 | 1378.8 | -3.5 | 0.0180832 | 0.692905 | Chlorophyll a-b binding protein 8, CAB8 |  |  |  |  |  |  |  |
| Solyc10g007320.2 | 30.5 | 315.9 | -3.4 | 0.00102664 | 0.190336 | Uroporphyrinogen decarboxylase 1 |  |  |  |  |  |  |  |
| Solyc12g094640.1 | 457.5 | 4407.6 | -3.3 | 0.0321221 | 0.814299 | Glyceraldehyde-3-phosphate dehydrogenase B, GAPB |  |  |  |  |  |  |  |
| Solyc12g099650.1 | 939.8 | 8443.6 | -3.2 | 0.0367732 | 0.835438 | Photosystem II 5 kDa protein, PSBT |  |  |  |  |  |  |  |
| Solyc12g009600.1 | 519.2 | 4362.8 | -3.1 | 0.000863636 | 0.181573 | Thylakoid lumenal 16.5 kDa protein |  |  |  |  |  |  |  |
| Solyc03g005260.2 | 99.4 | 766.3 | -2.9 | 0.000245734 | 0.0988108 | ATP sulfurylase 1, APS1 |  |  |  |  |  |  |  |
| Solyc06g064550.2 | 21.3 | 160.5 | -2.9 | 0.00238262 | 0.276172 | Bifunctional aspartokinase/homoserine dehydrogenase 1 |  |  |  |  |  |  |  |
| Solyc06g066640.2 | 246.7 | 1640.5 | -2.7 | 0.035219 | 0.817497 | Photosystem I reaction center subunit VI-1, PSAH1 |  |  |  |  |  |  |  |
| Solyc01g006330.2 | 15.8 | 104.7 | -2.7 | 0.0114225 | 0.584144 | Probable plastid-lipid-associated protein 12, PAP12 |  |  |  |  |  |  |  |
| Solyc06g084050.2 | 332.5 | 2200.5 | -2.7 | 0.0205993 | 0.720184 | Photosystem II reaction center W protein, PSBW |  |  |  |  |  |  |  |
| Solyc12g009200.1 | 230.1 | 1504.5 | -2.7 | 0.0317196 | 0.814299 | Chlorophyll a-b binding protein |  |  |  |  |  |  |  |
| Solyc06g007160.2 | 48.7 | 317.4 | -2.7 | 0.000819955 | 0.17797 | Probable NADH dehydrogenase ，DDB |  |  |  |  |  |  |  |
| Solyc03g082890.2 | 68.2 | 439.1 | -2.7 | 0.00588568 | 0.432542 | Thylakoid lumenal 17.4 kDa protein |  |  |  |  |  |  |  |
| S Solyc04g054520.2 | 43.4 | 275.4 | -2.7 | 0.00537033 | 0.4044 | Probable FKBP-type peptidyl-prolyl cis-trans isomerase 4 |  |  |  |  |  |  |  |
| Solyc03g063560.2 | 210.4 | 1316.1 | -2.6 | 0.0243046 | 0.75467 | Ferredoxin-dependent glutamate synthase 1,GLU1 |  |  |  |  |  |  |  |
| Solyc04g071960.2 | 12.7 | 78.3 | -2.6 | 0.0482303 | 0.903218 | Xanthoxin dehydrogenase ，ABA2 |  |  |  |  |  |  |  |
| Solyc01g087040.2 | 46.3 | 283.9 | -2.6 | 0.00399435 | 0.347331 | Thylakoid lumenal 19 kDa protein |  |  |  |  |  |  |  |
| Solyc02g069450.2, | 110.9 | 676.7 | -2.6 | 0.0313898 | 0.813799 | Photosystem I reaction center subunit III,PSAF |  |  |  |  |  |  |  |
| Solyc11g044250.1 | 1218.2 | 7347.0 | -2.6 | 0.00138617 | 0.216254 | K(^+^) efflux antiporter 3, KEA3 |  |  |  |  |  |  |  |
| Solyc06g083680.2 | 117.2 | 701.4 | -2.6 | 0.00880617 | 0.521283 | Photosystem I reaction center subunit IV A, PSAEA |  |  |  |  |  |  |  |
| Solyc03g019940.2 | 21.4 | 127.1 | -2.6 | 0.0146309 | 0.633881 | Asparagine-tRNA ligase,SYNO |  |  |  |  |  |  |  |
| Solyc09g065620.2 | 4.9 | 28.9 | -2.6 | 0.0150689 | 0.641557 | Chlorophyllase-2, CLH2 |  |  |  |  |  |  |  |
| Solyc08g077050.2 | 40.5 | 217.0 | -2.4 | 0.0112445 | 0.584071 | Ferredoxin-2 petF2 |  |  |  |  |  |  |  |
| Solyc05g009780.2 | 38.8 | 206.4 | -2.4 | 0.00203008 | 0.253597 | Methionine aminopeptidase 1B, MAP1B |  |  |  |  |  |  |  |
| Solyc04g009420.2 | 34.1 | 178.4 | -2.4 | 0.00513097 | 0.397184 | PsbP domain-containing protein 2, PPD2 |  |  |  |  |  |  |  |
| Solyc06g065490.2 | 39.5 | 199.7 | -2.3 | 0.011504 | 0.584144 | PsbP domain-containing protein 6, PPD6 |  |  |  |  |  |  |  |
| Solyc07g043570.2 | 42.6 | 214.7 | -2.3 | 0.0203418 | 0.718873 | Uncharacterized oxidoreductase At1g06690 |  |  |  |  |  |  |  |
| Solyc04g082920.2 | 17.4 | 87.4 | -2.3 | 0.0211918 | 0.729873 | Chlorophyll a-b binding protein of LHCII type I |  |  |  |  |  |  |  |
| Solyc10g075160.1 | 270.7 | 1348.3 | -2.3 | 0.0446707 | 0.875844 | Ferredoxin-1, SEND33 |  |  |  |  |  |  |  |
| Solyc08g067840.2 | 25.1 | 123.6 | -2.3 | 0.0231149 | 0.741764 | PsbP domain-containing protein 5, PPD5 |  |  |  |  |  |  |  |
| Solyc12g070270.1 | 5.5 | 27.1 | -2.3 | 0.00713708 | 0.475927 | ABC transporter B family member 26, ABCB26 |  |  |  |  |  |  |  |
| Solyc02g086910.2 | 118.7 | 574.7 | -2.3 | 0.00481469 | 0.380612 | Peptidyl-prolyl cis-trans isomerase CYP38, CYP38 |  |  |  |  |  |  |  |
| Solyc07g009170.2 | 7.4 | 35.7 | -2.3 | 0.0034521 | 0.329834 | Nudix hydrolase 20, NUDT20 |  |  |  |  |  |  |  |
| Solyc03g119150.2 | 26.6 | 127.6 | -2.3 | 0.00993031 | 0.553771 | Probable FKBP-type peptidyl-prolyl cis-trans isomerase 5 |  |  |  |  |  |  |  |
| Solyc11g006580.1 | 11.5 | 54.9 | -2.3 | 0.00363662 | 0.334071 | Nudix hydrolase 26 |  |  |  |  |  |  |  |
| Solyc08g006160.2 | 48.6 | 231.5 | -2.3 | 0.0363612 | 0.830755 | Probable plastid-lipid-associated protein 8, PAP8 |  |  |  |  |  |  |  |
| Solyc01g108210.2 | 4.9 | 23.5 | -2.2 | 0.0114872 | 0.584144 | Abscisic acid 8'-hydroxylase 4 ，CYP707A4 |  |  |  |  |  |  |  |
| Solyc02g087230.2 | 29.8 | 140.6 | -2.2 | 0.00738005 | 0.483393 | Ferredoxin-thioredoxin reductase catalytic chain,FTRC |  |  |  |  |  |  |  |
| Solyc08g067320.1 | 115.3 | 541.2 | -2.2 | 0.0329831 | 0.814933 | Chlorophyll a-b binding protein 25,CAB25 |  |  |  |  |  |  |  |
| Solyc03g114930.2 | 30.2 | 141.1 | -2.2 | 0.022415 | 0.734619 | PsbP-like protein 1, PPL1 |  |  |  |  |  |  |  |
| Solyc07g054290.1 | 245.1 | 1141.3 | -2.2 | 0.0415334 | 0.854722 | Thylakoid lumenal protein At1g03610 |  |  |  |  |  |  |  |
| Solyc03g006870.2 | 41.4 | 192.8 | -2.2 | 0.0157803 | 0.660416 | Phosphoglucomutase, =PGMP |  |  |  |  |  |  |  |
| Solyc04g015040.2 | 72.2 | 331.0 | -2.2 | 0.00529599 | 0.404303 | Probable FKBP-type peptidyl-prolyl cis-trans isomerase 2 |  |  |  |  |  |  |  |
| Solyc12g006810.1 | 27.9 | 127.7 | -2.2 | 0.0127899 | 0.602752 | Heme-binding-like protein At3g10130 |  |  |  |  |  |  |  |
| Solyc10g006040.1 | 33.5 | 151.7 | -2.2 | 0.0083651 | 0.513005 | Serine acetyltransferase 1,SAT1 |  |  |  |  |  |  |  |
| Solyc02g087400.1 | 61.6 | 278.9 | -2.2 | 0.0304532 | 0.8055 | Signal recognition particle 43 kDa protein,CAO |  |  |  |  |  |  |  |
| Solyc00g006810.2 | 27.9 | 125.3 | -2.2 | 0.0201366 | 0.718873 | Probable FKBP-type peptidyl-prolyl cis-trans isomerase 7 |  |  |  |  |  |  |  |
| Solyc04g015450.2 | 18.2 | 80.7 | -2.2 | 0.00933076 | 0.535165 | Translation factor GUF1 homolog |  |  |  |  |  |  |  |
| Solyc01g094340.2 | 12.5 | 54.5 | -2.1 | 0.0460383 | 0.883991 | Acetyl-coenzyme A carboxylase carboxyl transferase subunit alpha, CAC3 |  |  |  |  |  |  |  |
| Solyc08g075490.2 | 33.6 | 145.8 | -2.1 | 0.00605718 | 0.435906 | Probable carotenoid cleavage dioxygenase 4, CCD4 |  |  |  |  |  |  |  |
| Solyc03g026210.2 | 19.4 | 83.8 | -2.1 | 0.032722 | 0.814299 | Putative dihydrodipicolinate reductase 3, DAPB3 |  |  |  |  |  |  |  |
| Solyc03g019880.2 | 30.3 | 130.7 | -2.1 | 0.00649658 | 0.452289 | UPF0426 protein At1g28150 |  |  |  |  |  |  |  |
| Solyc10g006530.2 | 42.1 | 180.8 | -2.1 | 0.0101103 | 0.556794 | Oxygen-evolving enhancer protein 3-1, PSBQ1 |  |  |  |  |  |  |  |
| Solyc08g076480.2 | 28.9 | 122.6 | -2.1 | 0.023493 | 0.744886 | Plastoglobulin-1, PG1 |  |  |  |  |  |  |  |
| Solyc03g117850.2 | 12.2 | 51.1 | -2.1 | 0.00744168 | 0.485992 | Ribulose bisphosphate carboxylase/oxygenase activase |  |  |  |  |  |  |  |
| Solyc04g077130.1 | 83.2 | 346.7 | -2.1 | 0.0280059 | 0.788833 | 50S ribosomal protein 6,PSRP6 |  |  |  |  |  |  |  |
| Solyc10g047930.1 | 59.4 | 242.9 | -2.0 | 0.0259194 | 0.764441 | Peptide methionine sulfoxide reductase B2, MSRB2 |  |  |  |  |  |  |  |
| Solyc01g087520.2 | 99.2 | 398.1 | -2.0 | 0.0277031 | 0.78379 | Ferredoxin-thioredoxin reductase |  |  |  |  |  |  |  |
| Solyc03g120430.2 | 67.0 | 267.0 | -2.0 | 0.0257522 | 0.764441 | D-glycerate 3-kinase, GLYK |  |  |  |  |  |  |  |
| Solyc08g079820.2 | 9.2 | 36.6 | -2.0 | 0.0111632 | 0.581727 | Nudix hydrolase 14, NUDT14 |  |  |  |  |  |  |  |
| Solyc06g083690.2 | 41.9 | 166.0 | -2.0 | 0.00993031 | 0.553771 | Glutaredoxin-C5, GRXC5 |  |  |  |  |  |  |  |
| Solyc03g118240.2 | 28.8 | 113.2 | -2.0 | 0.0171724 | 0.688133 | Magnesium-protoporphyrin O-methyltransferase， chlM |  |  |  |  |  |  |  |
| Solyc12g015680.1 | 24.4 | 91.5 | -1.9 | 0.0153393 | 0.646849 | Protein TIC 20-v, TIC20-V |  |  |  |  |  |  |  |
| Solyc06g009220.2 | 14.5 | 54.1 | -1.9 | 0.0129982 | 0.602752 | Isoamylase 3, ISA3 |  |  |  |  |  |  |  |
| Solyc02g069010.2 | 49.1 | 182.0 | -1.9 | 0.0136599 | 0.617178 | Phosphatase IMPL1, IMPL1 |  |  |  |  |  |  |  |
| Solyc10g051110.1 | 123.0 | 451.5 | -1.9 | 0.0243862 | 0.756107 | Protein TIC 62, TIC62 |  |  |  |  |  |  |  |
| Solyc06g082760.2 | 52.9 | 192.3 | -1.9 | 0.025296 | 0.760907 | 50S ribosomal protein L17, RPL17 |  |  |  |  |  |  |  |
| Solyc01g091780.2 | 25.7 | 92.9 | -1.9 | 0.0395249 | 0.849556 | Protein-ribulosamine 3-kinase |  |  |  |  |  |  |  |
| Solyc07g005580.2 | 14.7 | 52.5 | -1.8 | 0.0201967 | 0.718873 | 1-acyl-sn-glycerol-3-phosphate acyltransferase 1, LPAT1 |  |  |  |  |  |  |  |
| Solyc08g006720.2 | 60.6 | 214.6 | -1.8 | 0.0186905 | 0.702526 | Putative glutathione peroxidase 7, GPX7 |  |  |  |  |  |  |  |
| Solyc08g015660.2 | 20.2 | 71.5 | -1.8 | 0.0193977 | 0.713363 | 6,7-dimethyl-8-ribityllumazine synthase |  |  |  |  |  |  |  |
| Solyc08g068570.2 | 3.8 | 13.4 | -1.8 | 0.021169 | 0.729873 | Tocopherol cyclase, VTE1 |  |  |  |  |  |  |  |
| Solyc04g071940.2 | 42.0 | 146.6 | -1.8 | 0.0429829 | 0.863806 | Xanthoxin dehydrogenase ，ABA2 |  |  |  |  |  |  |  |
| Solyc10g084040.1 | 44.9 | 155.4 | -1.8 | 0.0304305 | 0.8055 | Thylakoid lumenal 15.0 kDa protein 2 |  |  |  |  |  |  |  |
| Solyc08g005220.2 | 5.8 | 19.7 | -1.8 | 0.0202081 | 0.718873 | Probable plastid-lipid-associated protein 3, PAP3 |  |  |  |  |  |  |  |
| Solyc07g045540.2 | 33.7 | 113.3 | -1.8 | 0.0216771 | 0.73037 | Glucose-6-phosphate 1-dehydrogenase |  |  |  |  |  |  |  |
| Solyc11g044530.1 | 36.6 | 121.5 | -1.7 | 0.0370548 | 0.835438 | Probable plastid-lipid-associated protein 4, PAP4 |  |  |  |  |  |  |  |
| Solyc07g064940.2 | 27.8 | 92.2 | -1.7 | 0.0348679 | 0.817497 | Thioredoxin-like protein HCF164, HCF164 |  |  |  |  |  |  |  |
| Solyc01g079220.2 | 27.3 | 88.6 | -1.7 | 0.031173 | 0.813799 | NifU-like protein 1, NIFU1 |  |  |  |  |  |  |  |
| Solyc01g006450.2 | 37.3 | 120.5 | -1.7 | 0.0451171 | 0.880057 | Enoyl-[acyl-carrier-protein] reductase [NADH] |  |  |  |  |  |  |  |
| Solyc12g096590.1 | 39.0 | 125.9 | -1.7 | 0.0374368 | 0.838879 | 6,7-dimethyl-8-ribityllumazine synthase |  |  |  |  |  |  |  |
| Solyc04g082680.2 | 62.1 | 199.4 | -1.7 | 0.026655 | 0.764441 | Outer envelope pore protein 21B, OEP21B |  |  |  |  |  |  |  |
| Solyc06g073090.2 | 6668.7 | 20981 | -1.7 | 0.0396868 | 0.850149 | 30S ribosomal protein 1, RPS30 |  |  |  |  |  |  |  |
| Solyc10g078920.1 | 46.0 | 141.8 | -1.6 | 0.0346704 | 0.817497 | Thioredoxin-like 3-1, WCRKC1 |  |  |  |  |  |  |  |
| Solyc05g050980.2 | 30.4 | 93.3 | -1.6 | 0.0323668 | 0.814299 | 3-phosphoshikimate 1-carboxyvinyltransferase |  |  |  |  |  |  |  |
| Solyc02g064550.2 | 84.5 | 250.6 | -1.6 | 0.0378475 | 0.840202 | UPF0308 protein At2g37240 |  |  |  |  |  |  |  |
| Solyc01g080460.2 | 53.0 | 154.9 | -1.5 | 0.0416916 | 0.855431 | Pyruvate, phosphate dikinase, PPD |  |  |  |  |  |  |  |
| Solyc07g063570.2 | 20.5 | 57.4 | -1.5 | 0.0481066 | 0.903218 | Cytochrome c-type biogenesis ccda-like chloroplastic protein，CCDA |  |  |  |  |  |  |  |
