# Supplementary material for: Overexpression of SlRBZ Results in Chlorosis and Dwarfism through Impairing Chlorophyll, Carotenoid, and Gibberellin Biosynthesis in Tomato
Source: Front Plant Sci. 2016 Jun 22;7:907. doi: 10.3389/fpls.2016.00907 (PMC4916219; doi:10.3389/fpls.2016.00907)
Supplement: Table S2 — Primers sequences used for functional and expression analysis in this study. [file Table2.DOCX]

| **Table S2**. Primers sequences used for functional and expression analysis in this study. | |  |
| --- | --- | --- |
| primer name | forward primer sequence (5'-3') | reverse primer sequence (5'-3') |
| SlRBZ for subcellular localization | GCGGTACCATGGGACGAGAAGGAGATTGGGAGT | GCGGATCCTGCAACAGTAACAGGTTGAGTTGGT |
| *SlRBZ* for Q-PCR | GCACCTCCAGCTTCATCGTCTTC | GCGTCCTGCATTCAGCCTCTTGT |
| β-actin for Q-PCR | GTCCTCTTCCAGCCATCCAT | ACCACTGAGCACAATGTTACCG |
| *HEMA* for Q-PCR | GAGGACCGTCTTCGTAAAGC | GCACTTGTCCAGTTCAGCAA |
| *HEML* for Q-PCR | AAGAAAACCCATTGCAGCAC | AGACATGGAGCACCGAAACT |
| *HEMB1* for Q-PCR | CAGTTGTGCAAACAGGCAGT | GCTGAAATCCTTCAGCATCA |
| *HEMC* for Q-PCR | CAGGTTCCCTGGCTGAACT | CAACACATTGAGTGAGGGATAG |
| *HEME1* for Q-PCR | GTGGACAACTCCCACCTCAT | CCGTTCAAGAAGTCCACCAT |
| *HEMF1* for Q-PCR | CACCTAGGCAATGGTGGTTT | TCACAAGCACCTTTTTGCAC |
| *HEMG1* for Q-PCR | CATACTTTTCCACCTCCAAGCG | GTCAACTTCTGAGGGAGGAAC |
| *CHLD* for Q-PCR | GGAAGCGTGGAACAGCTAAA | AGCTAGCCCATCTTCCCACT |
| *CHLM* for Q-PCR | GACCGGTTGTTTGTCGATTC | ACTCCTCTTGCGCCTGTTT |
| *CRD* for Q-PCR | TCTCGCTTCTTCTGCCTTTC | AGTTGTGCGGTTTGTCTCAA |
| *DVR* for Q-PCR | TGTTTTGGTTGTGGGTTCAA | GCTCCATTTAACTGTTCCAAGGT |
| *PORA* for Q-PCR | CCATCTCGGACATTTCCTTC | ACCCTCCTGAAAGTCCTCGT |
| *CAO* for Q-PCR | CCTTTCTCTTTGTGCCGCTCT | CCTCCTCCCCATACACAGC |
| *CPS* for Q-PCR | ATACCTAGAGCTAGCGAAATC | ACTGCCTAAATAGTACGTAACC |
| *KS* for Q-PCR | TGATTTCTTTGATGTAGGAGGTTC | GCTTGCCACTTAGATGCTTTG |
| *KO* for Q-PCR | CCACGAAGACACGCAGGTAG | ATCGTTCAGGCTTCCACTCTT |
| *KAO* for Q-PCR | CTTTCAAATCCAACAATCCTG | TTAAAACCTTCCTGCAACCT |
| *GA2ox1* for Q-PCR | CTCATTTCTAATGCTCATCGT | TGCAGATGATTCTTTCTTA |
| *GA2ox2* for Q-PCR | TTTCCATATTCTACCCTACAAG | TCATCGCATTACAATACTCTT |
| *GA2ox5* for Q-PCR | GATCACTTACCAATAATCAACAG | CGTCATGGTTTACGACTTTA |
| *GA20ox2* for Q-PCR | TTTCCATATTCTACCCTACAAG | TCATCGCATTACAATACTCTT |
| *GA20ox4* for Q-PCR | GATGATAAATGGCACTCTATTC | TGACTTCCTTGTTCTTCTACAG |
| *PSY2* for Q-PCR | GCGCCTTCCATTGAAGCCAAGTAT | CCTTTGATTCAGGGGCGATACC |
| *PDS* for Q-PCR | TTGTGTTTGCCGCTCCAGTGGATAT | GCGCCTTCCATTGAAGCCAAGTAT |
| *ZDS* for Q-PCR | ATTATTACATTGAGGGACAAGGCT | TCATCAGACAAGACTCAACTCATC |
| *CrtR-b* for Q-PCR | GCGGATCCATGTTTAATAACCACCAGCACTTG | GCGGTACCGAGTGCATTTGCGGAAGTTAC |
| *LCY-B* for Q-PCR | GCGTCGACTTCAAGATGTTTAATAACCACCAGC | GCGTCGACACCTTTCATGCATTTGCGGATAC |
| *DXS* for Q-PCR | GCAGTCTTGGTGTTGTTGAG | GCTGTCCCTTCTACCAGTCAA |
| *DXR* for Q-PCR | GCGCACTTGCTGCTGGTTCA | GCAATCTCAGGCTTGTCTTCCAT |
| *GGPS* for Q-PCR | TATGCAGAAAACATATTACAAGA | ATCAAGAACATCATCTATTAATTG |
| *Solyc04g082680* for Q-PCR | GCCACTTCGTTATGCTGGAGATT | GCGTATTGCCTTATCATAGCACAGA |
| Full length *SlRBZ* for Y2H bait vector | GCCCCGGGTATGGGACGAGAAGGAGATTG | GCCTGCAGCTATGCAACAGTAACAGGTTGAG |
| Full length *SlRBZ* for BiFC | GCTCTAGAATGGGACGAGAAGGAGATTG | GCGGTACCTGCAACAGTAACAGGTTGAGTTGGT |
| Full length *Solyc04g082680* for BiFC | GCTCTAGAATGGAGAGCTCACTTCGTTATGC | GCGGTACCCCATTTCCCAATATTGTTGACAGTG |
| *SlRBZ* promoter | GCGAGCTCACCGCTGATGAGTAACAATGAAAC | GCCTCGAGTGTATGTTATCTTCTTCGTCTCTCCG |
